# Pre-regulation of the planar chirality of pillar[5]arenes for preparing discrete chiral nanotubes†

**DOI:** 10.1039/d1sc00074h

**Published:** 2021-02-03

**Authors:** Shixin Fa, Keisuke Adachi, Yuuya Nagata, Kouichi Egami, Kenichi Kato, Tomoki Ogoshi

**Affiliations:** Department of Synthetic Chemistry and Biological Chemistry, Graduate School of Engineering, Kyoto University Katsura, Nishikyo-ku Kyoto 615-8510 Japan ogoshi@sbchem.kyoto-u.ac.jp; WPI Institute for Chemical Reaction Design and Discovery, Hokkaido University Kita 21 Nishi 10, Kita-ku Sapporo 001-0021 Japan; WPI Nano Life Science Institute (WPI-NanoLSI), Kanazawa University Kakuma-machi Kanazawa Ishikawa 920-1192 Japan

## Abstract

Regulating the chirality of macrocyclic host molecules and supramolecular assemblies is crucial because chirality often plays a role in governing the properties of these systems. Herein, we describe pillar[5]arene-based chiral nanotube formation *via* pre-regulation of the building blocks' chirality, which is different from frequently used post-regulation strategies. The planar chirality of rim-differentiated pillar[5]arenes is initially regulated by chiral awakening and further induction/inversion through stepwise achiral external stimuli. The pre-regulated chiral information is well stored in discrete nanotubes by interacting with a per-alkylamino-substituted pillar[5]arene. Such pre-regulation is more efficient than post-regulating the chirality of nanotubes.

## Abstract 2

Pillar[5]arene-based chiral nanotube formation *via* pre-regulation of the building blocks' chirality is more efficient than post-regulating the chirality of nanotubes.

## Graphical abstract

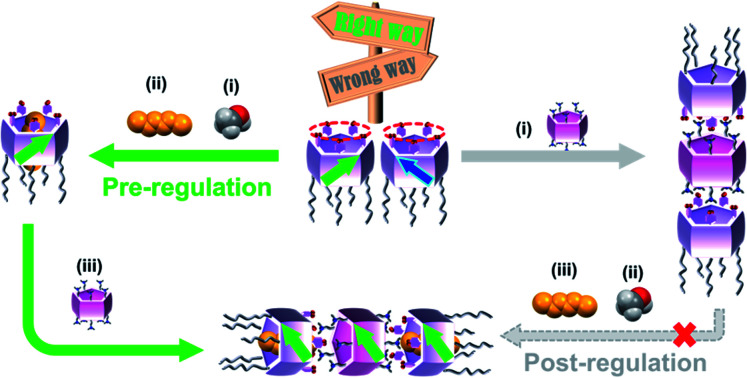

## Introduction

Chirality is widely observed in nature and critically important to living things and life processes;^1^ this property has also been applied extensively in the field of materials science.^2^ Research efforts have continuously focused on controlling the chirality of supramolecular assemblies^3,4^ or polymers^5,6^ because chirality often has significant effects on the properties of these artificial systems, as observed in biological systems and applications.^7^ Chiral control is often obtained using post-regulation strategies, meaning that the chirality (*e.g.*, helicity) of the materials is regulated *via* external stimuli after generating the desired supramolecular assemblies or polymers depending on the kinetic stability of these materials.^4,6,8^ Another useful strategy is pre-regulation, wherein the chirality of the building blocks is regulated, and the assemblies or polymers are constructed using those chiral building blocks. However, acute control of the chirality of building blocks remains underexplored,^9,10^ because of the complexity and subtlety of molecule design. In especial, chiral control of macrocycles,^11^ which are of critical importance in supramolecular chemistry as host molecules or building blocks of supramolecular assemblies, are rarely reported. Common methods in this regard are asymmetric syntheses^11*a*^ and chiral isolation using HPLC.^11*b*–*d*^ Acute control of the chirality (*e.g.*, planar chirality and inherent chirality) of macrocycles that can be used as building blocks through post-synthesis regulations has not been explored. Herein, we report a pillar[5]arene-based chirality control system, in which the planar chirality of building block molecules is governed by stepwise addition of external stimuli (taking a planar chiral racemic mixture to either *pS*- or *pR*-rich products). The pre-regulated chiral pillar[5]arenes are then employed for discrete chiral nanotube formation.

**Fig. 1:**
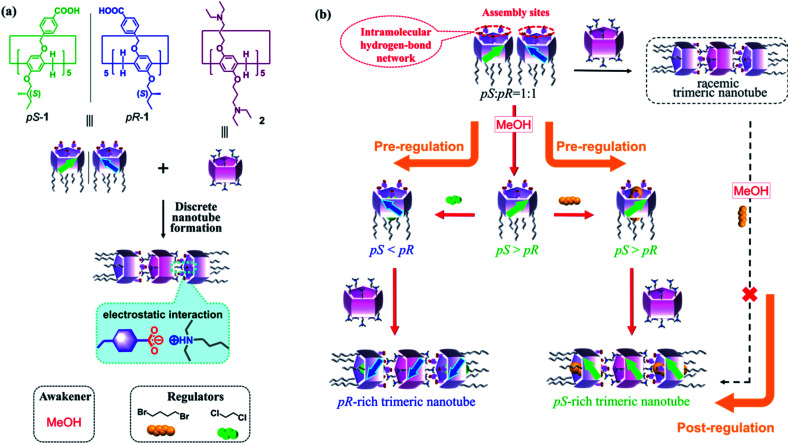
(a) Molecular structures and discrete nanotube formation. (b) Multi-step pre-regulation of the planar chirality of 1, and preparation of discrete chiral nanotubes. The black solid and dashed arrows illustrate a post-regulation pathway, which is unsuccessful to prepare chiral nanotubes.

Pillar[*n*]arenes, which were first reported by our group in 2008,^12^ have attracted attention as macrocyclic host molecules in supramolecular chemistry.^13,14^ In general, pillar[*n*]arenes possess two planar chiral isomers (*i.e.*, *pS* and *pR*, Fig. 1a), which can interconvert in solution *via* rotation of the units depending on the bulkiness of the two rims.^13,14*d*,*f*,15^ Introducing bulky alkoxy substituents is necessary to inhibit the rotation of the unit, and separate these enantiomers by chiral column chromatography^16^ or formation of diastereomers *via* chiral reagents.^17^ In addition, the planar chirality of pillar[5]arenes can be influenced by achiral solvents or guest molecules interacting with chiral substituents on the rims.^18,19^ For example, our previous work showed that the planar chirality of a pillar[5]arene containing ten 2-(*S*)-methylbutoxy groups was maintained or inverted based on the linear dihaloalkane solvent used.^18*a*^ However, such one-factor regulation was not used for further assembly due to lack of interaction sites in the molecule. In the current work, we designed pillar[5]arene 1 with five 2-(*S*)-methylbutoxy groups on one rim and five benzoic acid groups on the other (Fig. 1a). The rim with chiral substituents induced the planar chirality of 1 and took on the role for chiral regulation, while the rim with benzoic acid groups served to provide interaction sites to guarantee supramolecular assembly.^20^ Moreover, taking the advantage of the five benzoic acids on an identical rim, the planar chirality of 1 was initially unexpressed due to the formation of intramolecular hydrogen bond network (HBN),^4*d*^ making the solution a planar chiral racemate. The planar chirality of 1 was then regulated by a series of achiral external stimuli, including (i) planar chiral awakening by cutting the HBN with methanol (MeOH), and (ii) further induction or inversion of planar chirality with dihaloalkane guests (Fig. 1b). The pre-regulated chiral information of 1 can be perfectly stored in discrete trimeric nanotubes developed based on electrostatic interactions with peralkylamino-substituted pillar[5]arene 2. To the best of our knowledge, this is the first time that a pre-regulation strategy has been applied to obtain discrete chiral nanotubes based on pillar[5]arenes. Post-regulation of such nanotubes, which would involve trimer formation followed by addition of the awakener and regulator, would not work well in this system.

## Results and discussion

### Synthesis and CD silencing

**Fig. 2:**
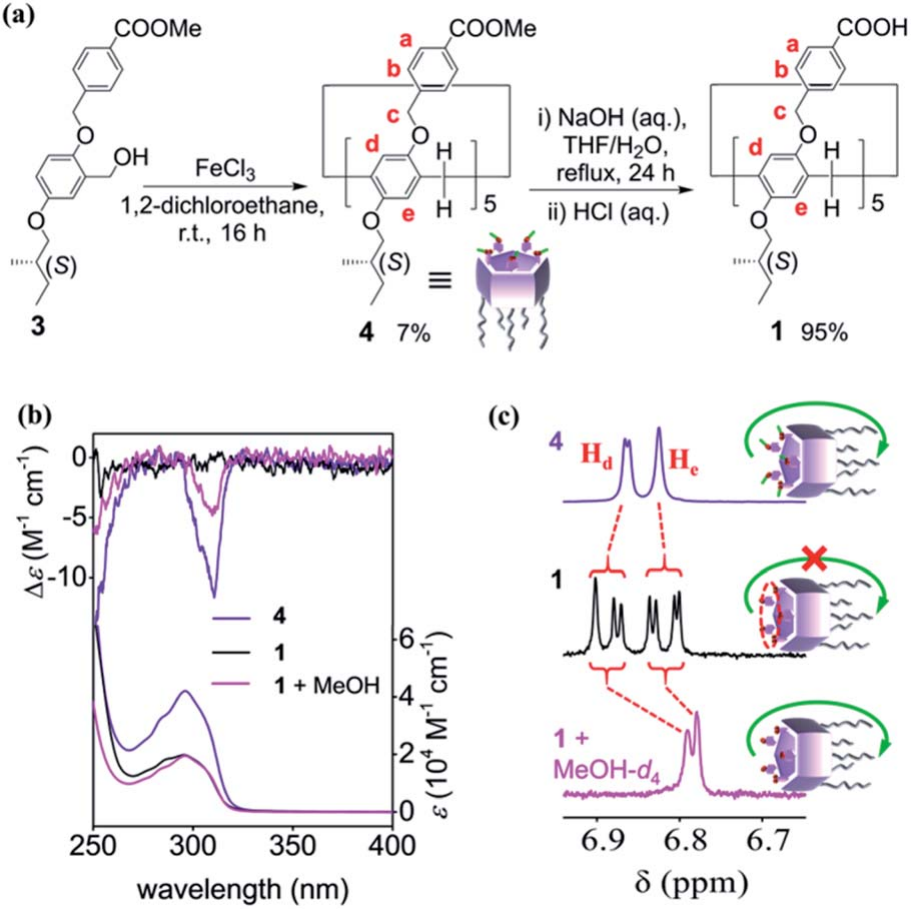
(a) Synthetic route to 1. Only the *pS* forms of 1 and 4 are shown. (b) UV-vis and CD spectra (0.1 mM) in chloroform and (c) partial ^1^H NMR spectra (0.1 mM, 600 MHz) of 1 and 4 in CDCl_3_ at 20 °C. In case of 1, the spectra with 10% of MeOH or MeOH-d_4_ are also shown.

Fig. 2a illustrates the synthetic procedure for the rim-differentiated pillar[5]arene 1. The disubstituted monomer, 3, which was prepared by stepwise S_N_2 substitutions of 2,5-dihydroxybenzaldehyde followed by the aldehyde reduction (Scheme S1†), was cyclized in the presence of FeCl_3_ in 1,2-dichloroethane to produce rim-differentiated pillar[5]arene 4.^21^ Hydrolysis of the ester groups on one rim of 4, using NaOH in a mixture of water and tetrahydrofuran, generated compound 1 in 95% yield.

In the UV-vis spectra, both 1 and 4 have a broad absorption band at around 296 nm (Fig. 2b), which is ascribed to π–π* transitions in the pillar[5]arene backbone (Fig. S1†).^18*b*^ Additionally, a negative Cotton effect was observed in the π–π* transition region of the circular dichroism (CD) spectrum of 4 in chloroform (purple line in Fig. 2b), indicating that *pS* planar chirality of 4 was self-induced by the stereogenic carbons on the rim.^19*b*^ In contrast, CD signal was silenced after hydrolyzing the ester groups of 4, as no Cotton effect was observed for 1 (black line in Fig. 2b). Heating the sample in chloroform to 60 °C and keeping it for 5 min, no self-induction was detected, indicating that kinetically stable racemic structures of 1 were formed in chloroform (Fig. S2†).

The proton signals of 1 were split into four sets in the ^1^H NMR spectrum in chloroform-d_1_ (CDCl_3_),^22^ which was more complicated than the spectrum of 4 (purple and black lines in Fig. 2c and S4†), indicating that the unit rotation of 1 was suppressed, and the rotation speed of the units was slower than the NMR time scale.^15^ The spectrum of 1 barely changed even if the sample was heated to 60 °C (Fig. S5†). In other nonpolar solvents, such as dichlorometane-d_2_, the ^1^H NMR spectrum of 1 was as complicated as that in CDCl_3_ (Fig. S6†). However, the ^1^H NMR spectra of 1 show only one set of peaks in MeOH-d_4_ and acetone-d_6_. Considering that MeOH-d_4_ and acetone-d_6_ can efficiently break hydrogen bonds, the differing spectroscopic behavior between 4 and 1 probably originated from the intramolecular HBN of 1.

Unfortunately, we could not obtain single crystals of 1. However, intermolecular HBN mediated by water molecules was observed clearly between two pillararenes in the crystal of dimeric structure constructed from an analogue of 1,^20^ which confirmed the ability of benzoic acid groups in 1 for HBN formation. Density functional theory (DFT) calculations were conducted to further understand the structures of 1. The HBN between five benzoic acid moieties mediated by five water molecules was found to significantly stabilize the structure of 1 by decreasing the free energy of 53.8 kJ mol^−1^ at ωB97XD/6-31G(d,p) level when comparing with the structure of 1 without HBN (Fig. S7†).

It is probable that the two diastereomers of 1 were structurally fixed *via* HBN in a ratio of 1 : 1 in chloroform, making the self-induction of planar chirality of 1 difficult. Therefore, HBN-breaking is necessary for CD signal self-induction.

### Awakening of CD signal

As MeOH-d_4_ was gradually added to the solution of 1 in CDCl_3_, the complicated peaks in ^1^H NMR spectrum merged to some extent, such that ultimately, only one set of peaks was observed (pink line in Fig. 2c and S8†). Addition of MeOH increased the unit rotation of 1 by cutting the water-mediated intramolecular HBN, and further awakened its planar chirality, as confirmed by the negative CD signal (pink line in Fig. 2b).^23^ The self-induced planar chirality of 1 upon addition of MeOH was similar to that of 4. In fact, the asymmetry factor (*g* = Δ*ε*/*ε*) of chirality-awakened 1 at 310 nm was 83% that of 4, indicating that MeOH functioned as an awakener to initiate the chiral self-induction of 1.

Different from the observations in the CD-silenced solution of 1, heating the solution of 1 in the presence of MeOH caused gradual decrease of the awakened CD intensity (Fig. S9†). The CD intensity restored by cooling the sample to room temperature. These observations suggested that the suppression of unit rotation of 1 was eliminated by cutting the HBN *via* MeOH.

### Further induction and inversion of planar chirality

**Fig. 3:**
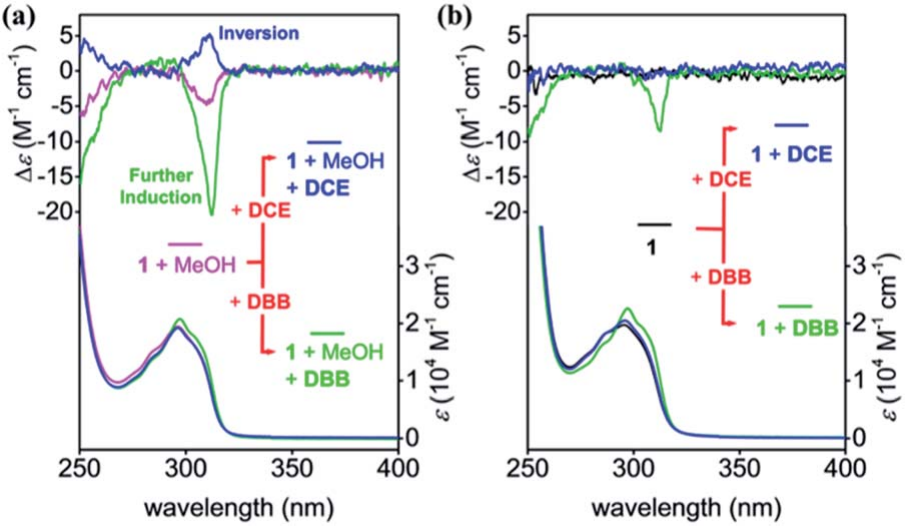
CD and UV-vis spectra of 1 (0.1 mM) with (a) and without (b) 10% MeOH in chloroform upon addition of DBB (5 equiv.) or DCE (35 equiv.) at 20 °C.

Guest inclusion plays an important role in the chiral induction of pillar[5]arenes with stereogenic carbon centers.^15,18,19^ Further *pS* induction was achieved when 5 equiv. 1,4-dibromobutane (DBB), which is an ideal guest molecule for the pillar[5]arene cavity (*K* > 10^3^ M^−1^),^13,24^ was added to the chirality-awakened solution of 1. This was revealed by the 4-fold increase of the CD intensity (green line in Fig. 3a), which indicated that the addition of DBB amplified the expression of planar chirality of 1. The diastereomeric excess (de) reached 20% after the amplification (Fig. S11†).

When a shorter linear guest, 1,2-dichloroethane (DCE), was added to the chirality-awakened solution of 1, a weak *pR* induction was observed (blue line in Fig. 3a), signaling the conversion of the planar chirality of 1. The difference between the induced chiral regulation of the chirality-awakened solution of 1 upon addition of DBB*versus*DCE is likely attributed to the arrangement of the stereogenic carbons on the rims of the respective guest complexed with 1, which is similar to the observation in our previous report.^18*a*^ The steric hindrance between the bromine atoms of DBB and aliphatic chains of 1 made the bulky ethyl branch on aliphatic chains out of the cavity, which resulted in a tendency of further *pS* induction of 1. While in the DCE case, the shorter guest made enough space in the cavity for the ethyl branch on aliphatic chains, leading a tendency of *pR* form of 1 (details in Fig. S12†).

Interestingly, chiral induction was not as successful when guest molecules were added directly to a solution of 1 without awakening the planar chirality *via* MeOH in advance (Fig. 3b, S13 and S14†). Addition of DBB induced around 30% of the CD intensity of the CD-silenced 1, compared with the chirality-awakened 1 (green line in Fig. 3b), while DCE failed entirely to induce planar chirality of 1 in the absence of MeOH (blue line in Fig. 3b). These observations suggest that chirality-awakening of 1 with MeOH was essential to further tune its planar chirality. Such multi-component control of planar chirality in macrocyclic host molecules in a single system is rarely reported, especially when considering that planar chirality was awakened from the racemate using achiral stimuli.

It is clear that the expression of the planar chirality of 1 depends on the external achiral stimuli, although the molecule contains five stereogenic carbons on its rim. However, both the awakener and the linear regulator demonstrated low efficiency for the chiral self-induction of 1 when used alone. The pre-regulation strategy by combined action of the awakener and linear regulator allows efficient control of the planar chirality of initially-racemic 1, to generate *pS*- or *pR*-rich forms (Fig. 1b). Utilizing the advantageous benzoic acid groups on one rim, 1 can produce trimeric nanotubes with *pS*- or *pR*-rich chirality by associating with 0.5 equiv. of per-alkylamino-substituted pillar[5]arene (2) *via* electrostatic interactions between the ionized amino groups and deprotonated benzoic groups (Fig. 1a).^20,25^

### Preparation of chiral nanotubes

**Fig. 4:**
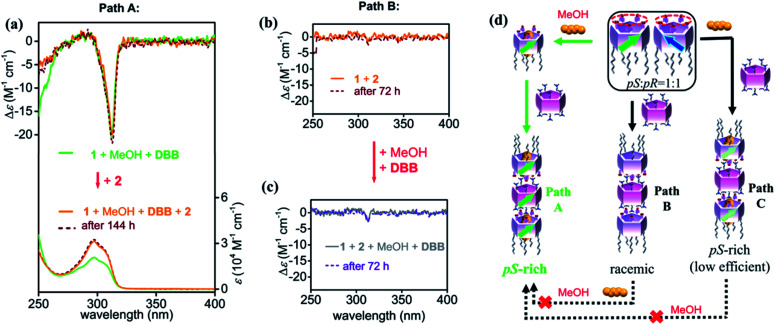
Trimeric nanotubes formation in various paths. (a) CD and UV spectral changes upon adding 2 (0.5 equiv.) in the mixture 1 (0.1 mM) + MeOH + DBB. CD spectra of (b) the mixture of 1 (0.1 mM) and 2 (0.5 equiv.) and (c) upon adding MeOH (10% volume fraction of the solution) and DBB (5 equiv.). (d) Illustration of *pS*-rich nanotubes obtained using pre- (green) and post-regulation (black) strategies.

Upon addition of 0.5 equiv. of 2 to the aforementioned pre-regulated solutions of 1 (*i.e.*, 1 + MeOH + DBB, and 1 + MeOH + DCE), trimeric nanotubular structures were formed and characterized by NMR spectroscopy and mass spectrometry measurements (Fig. S15–S17†). The CD intensity kept after addition of 2 (path A: orange line in Fig. 4a), indicating that the pre-regulated chiral character of 1 was perfectly stored upon formation of the *pS*-rich trimers. Upon heating the sample at 60 °C, the CD intensity was slightly decreased by *ca.* 15%, which was recovered after cooling to room temperature (Fig. S18†). In addition, no racemization was observed in 144 hours (dashed line in Fig. 4a), suggesting that such chiral trimers were kinetically stable due to the ten pairs of salt bridges. *pR*-Rich trimers were also obtained by adding 2 in the mixture 1 + MeOH + DCE (Fig. S19†). To the best of our knowledge, this is the first time discrete *pS*-rich and *pR*-rich nanotubes based on pillar[5]arenes with specific length and diameter have been obtained (possible nanotubular structures shown in Fig. S20†).

Without pre-regulation, trimeric nanotubes prepared from a mixture of 1 and 2 did not show any CD signal (path B: orange line in Fig. 4b), suggesting again that the trimeric nanotubes would well store the chiral information of 1. When the planar chirality of 1 was not self-induced by adding MeOH or DBB, the constructed trimeric nanotubes would also be racemic. The ten pairs of salt bridges made the racemic nanotubes very stable that no CD change was observed in 72 hours (dashed line in Fig. 4b). Interestingly, post-regulation had a weaker effect on the chirality of the nanotubes than pre-regulation (Fig. 4c). Successive addition of MeOH and DBB to the mixture of 1 and 2 caused slight increase of CD intensity, which was held in 3 days. Even if the sample was heated to 60 °C, no further induction was observed (Fig. S21†).

In the case where the chirality of 1 was not completely pre-regulated (*i.e.*, 1 + DBB in Fig. 3b), addition of 2 produced *pS*-rich trimers with lower CD intensity than that of path A by fixing the chiral information of 1 (Path C: Fig. S22a†), suggesting that the synergy of the awakener and linear regulator is essential to maximize the planar chirality of trimers. The additional external factor (*i.e.*, MeOH) hardly influenced the chirality of the ultimate trimers (Fig. S22b†). The pre-regulation strategy also worked well for preparing *pR*-rich nanotubes compared with the post-regulations (Fig. S23–S26†).

Although the ultimate components were same in paths A, B and C, it is clear that the CD intensities were different even after long-time keeping and heating. These observations indicated that the trimeric nanotubes constructed from benzoic acid-substituted pillar[5]arene 1 and per-alkylamino-substituted pillar[5]arene 2 were with high kinetic stability due to the ten pairs of salt bridges. Therefore, pre-regulation of the chirality of 1 through the combined action of the awakener (MeOH) and linear regulator (DBB or DCE) was essential for producing trimeric nanotubes with high degrees of chirality (Fig. S27†). This type of sequential strategy may be important for generating other highly stable assemblies.

**Fig. 5:**
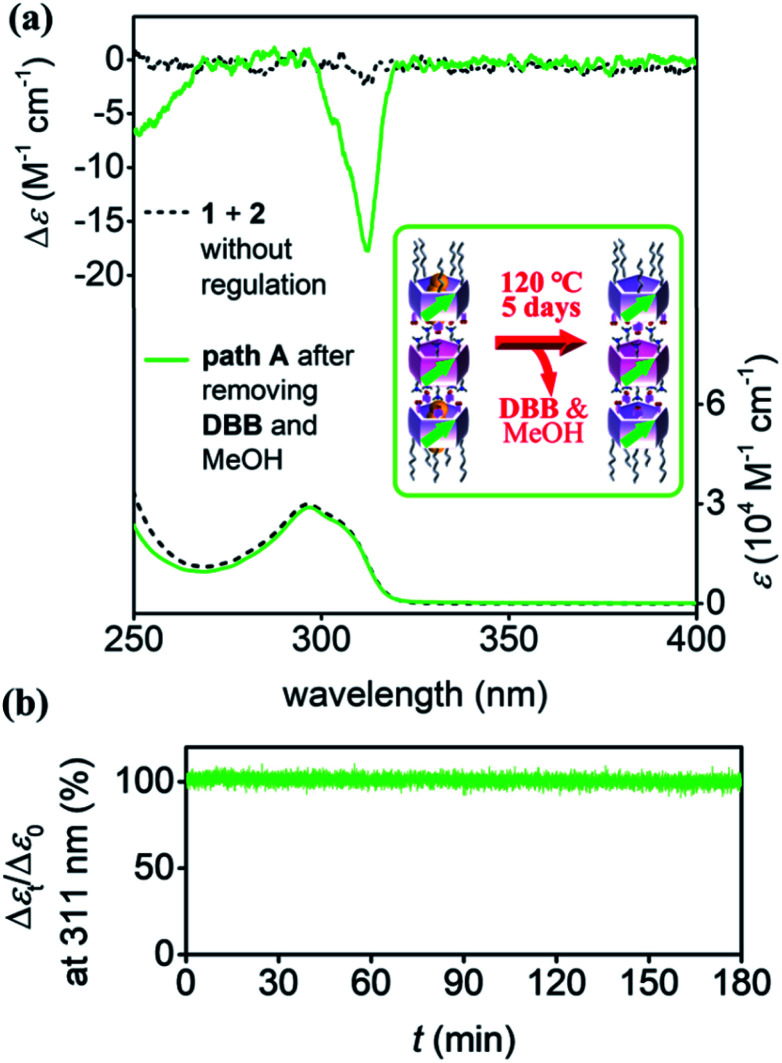
(a) CD and UV-vis spectra of hollow trimeric nanotubes (0.05 mM) prepared by mixing 1 and 2 (black lines), and by removing DBB and MeOH of the *pS*-rich nanotubes constructed *via* pre-regulation (green lines). (b) CD change of trimeric nanotubes (0.05 mM) at 311 nm after removing DBB and MeOH. The spectra were recorded at 20 °C in chloroform.

The *pS*-rich nanotubes formed *via* pre-regulation were dried under vacuum at 120 °C for 5 days to completely remove the MeOH and DBB from the solution and cavities of pillar[5]arenes (Fig. S28†). The planar chirality of the nanotubes was maintained after drying (green lines in Fig. 5a), and the CD intensity was much larger than that of the nanotubes prepared directly by mixing 1 and 2 (black lines). The planar chirality of the stepwise synthesized hollow nanotubes was stable in solution, even after removing MeOH and DBB. This was revealed by the time-dependent CD measurements; after 3 hours, there was only a negligible decrease in the CD intensity (Fig. 5b). Even after 1 month, the CD intensity decreased by only *ca.* 20% (Fig. S29†), indicating that the awakened planar chirality of 1 was maintained in the trimeric nanotubes.

## Conclusions

In conclusion, we prepared a unique system, in which the planar chirality expression of rim-differentiated pillar[5]arene 1 was initially silenced by an water-mediated intramolecular HBN, and then awakened and regulated by the combined action of MeOH and a linear guest (DBB or DCE). This multi-component planar chirality expression enabled control over the planar chirality of 1, from a racemic mixture to either *pS*- or *pR*-rich forms. The chiral information of 1 was ultimately stored in nanotubes following trimerization with per-alkylamino-substituted pillar[5]arene 2. Pre-regulation of the planar chirality of pillar[5]arene 1 was essential for creating chiral nanotubes. In contrast, post-regulating the chirality (after nanotube formation) was an ineffective approach to chiral regulation. Employing the novel process described herein, we obtained discrete chiral nanotubes with specific length and diameter. Preparation of chiral nanotubes is important in supramolecular chemistry to capture and store chiral guest molecules; future research efforts will aim to improve the planar chirality of rim-differentiated pillar[5]arenes *via* molecular design and to apply pre-regulation strategies to prepare nanotubes with higher diastereomeric excess.

## Conflicts of interest

There are no conflicts to declare.

## Supplementary Material

SC-012-D1SC00074H-s001
