# Supplementary material for: Pre-regulation of the planar chirality of pillar[5]arenes for preparing discrete chiral nanotubes
Source: Chem Sci. 2021 Feb 3;12(10):3483–8. doi: 10.1039/d1sc00074h (PMC8179476; doi:10.1039/d1sc00074h)
Supplement: SC-012-D1SC00074H-s001 [file SC-012-D1SC00074H-s001.pdf]

Electronic Supplementary Material (ESI) for Chemical Science.
This journal is © The Royal Society of Chemistry 2021

Electronic Supplementary Information

Pre-regulation of the Planar Chirality of Pillar[5]arenes for Preparing Discrete Chiral Nanotubes

Shixin Fa, Keisuke Adachi, Yuuya Nagata, Kouichi Egami, Kenichi Kato, and Tomoki Ogoshi*

Table of Contents

General………………………………………………………………………………...1

Syntheses…..................…………………………………………………………...…....1

CD silencing of 1...……...…5

CD awakening of 1...……...…9

Chirality regulation of 1 by DBB and DCE...........................……..……….....11

Nanotube formation of 1 and 2…..…14

Chirality storage of the nanotubes after drying……………......…..…......20

Supplementary discussion…..….....21

References………………………………………………………………………...…....22

^{1}H & ^{13}C NMR Spectra………………………………………………..…....23

General

All commercially available reagents and solvents were used as received. ^{1}H NMR and ^{13}C NMR spectra were recorded on JEOL JNM-ECS400, JNM-ECZ500R and JNM-ECA600P spectrometers at room temperature. Chemical shifts were reported in ppm versus tetramethylsilane. COSY and NOESY experiments were performed on JEOL JNM-ECA600P spectrometer at 30 °C. UV-vis absorption spectra and circular dichroism (CD) spectra were recorded at 20 °C on JASCO V-750 spectrophotometer and JASCO J-1500 CD spectrometer, respectively. 2 mm quartz cuvets were used for both UV-vis and CD measurements. High-resolution ESI-MS was recorded on Thermo Fisher Scientific Exactive Plus mass spectrometer equipped with UltiMate 3000 HPLC. DFT calculations for structures of **1** with and without intramolecular hydrogen bond network (HBN) were carried out at ωB97XD/6-31G(d,p) level of theory using Gaussian 16 Rev C.01.[1] The initial geometry was generated according to the single crystal X-ray diffraction analysis of a pillar[5]arene having benzoic acid moieties and *n*-butyloxyl chains and the addition of water molecules.[2]

Syntheses

Synthesis of peralkylamino-substituted pillar[5]arene **2**[2] and compound **5**[3] was followed reported procedure with optimization.

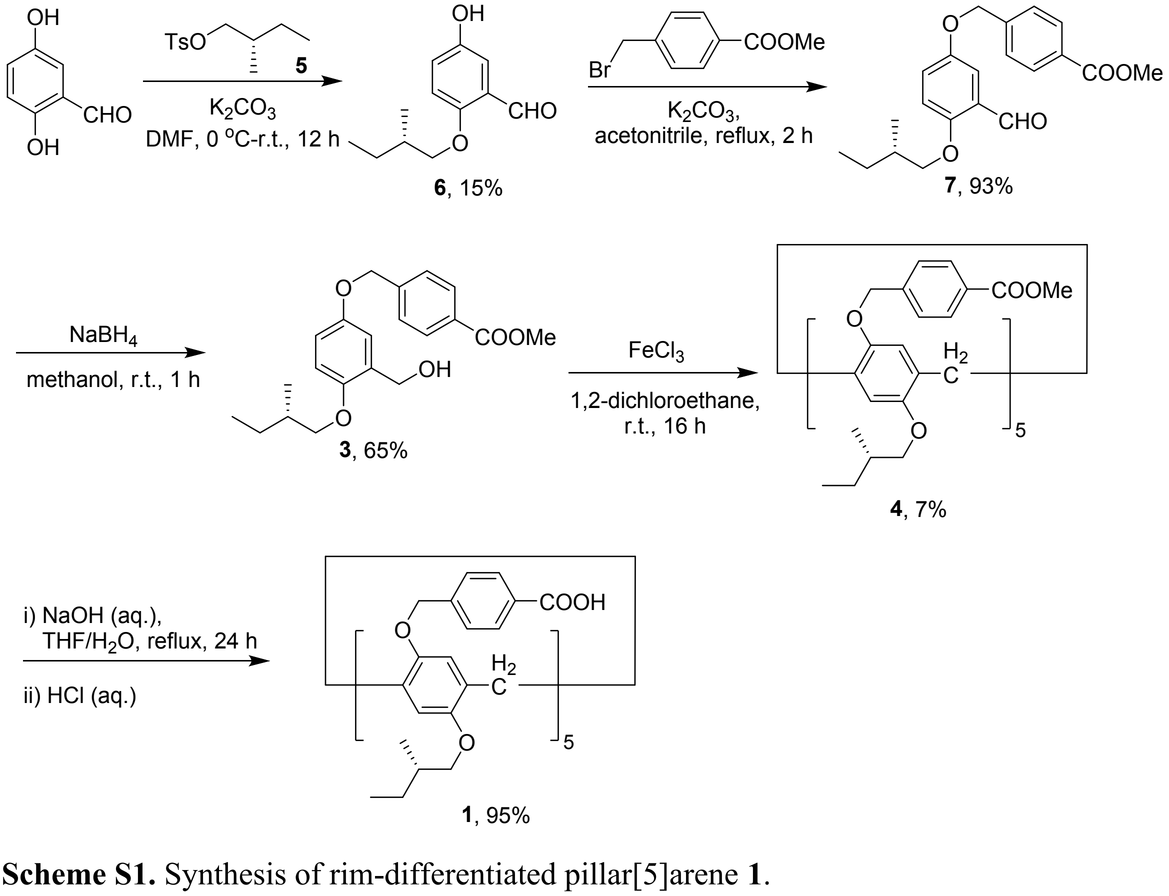

Scheme S1. Synthesis of rim-differentiated pillar[5]arene **1**.

Compound 6. To a mixture of tosylate **5** (17.5 g, 72.4 mmol) and potassium carbonate (20.0 g, 145 mmol) in DMF (100 mL) under 0 ^{o}C was added 2,5-dihydroxybenzaldehyde (10.0 g, 72.4 mmol). The mixture was stirred in ice bath for another 1 h, and then was allowed to stir at room temperature for 12 h. The solid was filtered off, and the filtrate was concentrated under reduced pressure. The residue was dissolved in ethyl acetate (450 mL), washed with brine (50 mL), and dried over sodium sulfate. The solid was filtered off. After removal of the solvent, the residue was chromatographed on a silica gel column using a mixture of *n*-hexane and ethyl acetate (from 10:1 to 5:1, v/v) as the mobile phase. Compound **6** was obtained as yellow oil (2.20 g, 10.6 mmol, 15%). ^{1}H NMR (500 MHz, $CDCl_3$): δ 10.46 (s, 1H), 7.34 (d, J = 2.8 Hz, 1H), 7.09 (dd, J = 3.5, 3.0 Hz, 1H), 6.90 (d, J = 3.5 Hz, 1H), 5.31 (s, 1H), 3.90 (dd, J = 6.0, 9.0 Hz, 1H), 3.81 (dd, J = 5.5, 8.5 Hz, 1H), 1.91 (m, 1H), 1.57 (m, 1H), 1.31 (m, 1H), 1.04 (d, J = 7.0 Hz 2H), 0.96 (t, 3H). ^{13}C NMR (125 MHz, $CDCl_3$): δ 190.0, 156.6, 149.5, 125.3, 123.6, 114.4, 113.4, 73.9, 34.9, 26.2, 16.7, 11.4. ESI-HRMS. Calcd for $C_{12}H_{16}O_3$ (m/z): $[M + H]^+$, 209.1172. Found: 209.1171.
Compound 7. To a stirred solution of mono-substituted dihydroxybenzaldehyde **6** (4.79 g, 23.0 mmol) in acetonitrile (230 mL) was added methyl 4-(bromomethyl) benzoate (5.27 mg, 23.0 mmol) and potassium carbonate (3.82 g, 27.6 mmol). The reaction mixture was stirred under reflux for 2 h. After cooled down to room temperature, the solid was filtered off, and the filtrate was concentrated under reduced pressure. The residue was chromatographed on a silica gel column using a mixture of *n*-hexane and acetone (from 10:1 to 5:1, v/v) as the mobile phase. Compound **7** was obtained as yellow oil (7.63 g, 21.0 mmol, 93%). ^{1}H NMR (400 MHz, $CDCl_3$): δ 10.50 (s, 1H), 8.05 (d, J = 8.0 Hz, 2H), 7.50 (d, J = 8.0 Hz, 2H), 7.40 (d, J = 3.6 Hz, 1H), 7.19 (dd, J = 3.4, 9.0 Hz, 1H), 6.94 (d, J = 9.2 Hz, 1H), 5.11 (s, 2H), 3.92 (s, 3H), 3.91-3.81 (m, 2H), 1.92 (m, 1H), 1.63-1.52 (m, 1H), 1.37-1.25 (m, 1H), 1.05 (d, J = Hz, 3H), 0.96 (t, 3H). ^{13}C NMR (125 MHz, $CDCl_3$): δ 189.6, 166.9, 156.9, 152.3, 142.0, 130.0, 129.8, 127.1, 125.2, 124.4, 114.4, 111.5, 73.9, 70.0, 52.2, 34.9, 26.2, 16.7, 11.4. ESI-HRMS. Calcd for $C_{21}H_{24}O_5$ (m/z): $[M + Na]^+$, 379.1516. Found: 379.1523.
Compound 3. To a stirred solution of di-substituted dihydroxybenzaldehyde **7** (7.63 g, 21.0 mmol) in methanol (70.0 mL) at 0 °C, sodium borohydride (976 mg, 25.7 mmol) was carefully added. The ice bath was removed, and the mixture was stirred at room temperature for 1 h. Brine (50 mL) was carefully added to quench the reaction. The mixture was extracted with dichloromethane (2 × 50 mL). The organic phase was combined and washed with water (50 mL) and brine (50 mL), and dried over anhydrous sodium sulfate. The solid was then filtered off. The filtrate was concentrated under reduced pressure to give compound **3** obtained as yellow oil (4.86 g, 13.5 mmol, 65%). ^{1}H NMR (400 MHz, $CDCl_3$): δ 8.04 (dd, J = 6.6, 1.8 Hz, 2H), 7.49 (d, J = 8.4 Hz, 2H), 6.96 (d, J = 2.7 Hz, 1H), 6.83-6.76 (m, 2H), 5.08 (s, 2H), 4.67 (d, J = 6.4 Hz, 2H), 3.92 (s, 3H), 3.85-3.73 (m, 2H), 2.37 (t, J = 6.5 Hz, 1H), 1.90-1.83 (m, 1H), 1.60-1.50 (m, 1H), 1.35-1.24 (m, 1H), 1.03 (d, J = 6.8 Hz, 3H), 0.95 (t, J = 7.5 Hz, 3H). ^{13}C NMR (100 MHz, $CDCl_3$): δ 167.1, 152.5, 151.7, 142.7, 130.6, 130.0, 129.8, 127.1, 115.9, 114.3, 112.1, 73.4, 70.2, 62.4, 52.3, 35.0, 26.4, 16.9, 11.5. ESI-HRMS. Calcd for $C_{21}H_{26}O_5$ (m/z): $[M + Na]^+$, 381.1672. Found: 381.1676.
Compound 4. To a stirred solution of di-substituted dihydroxybenzyl alcohol **3** (1.62 g, 4.50 mmol) in 1,2-dichloroethane (720 mL) was added anhydrous iron (III) chloride (77.0 mg, 0.473 mmol). The mixture was stirred at room temperature for 16 h before methanol (100 mL) was

added to quench the reaction. After concentrated under reduced pressure, the residue was chromatographed on a silica gel column using a mixture of *n*-hexane and acetone (10:1, v/v) as the mobile phase. The obtained white solid was further purified by thoroughly washing with a mixture of *n*-hexane and 2-propanol (99:1, v/v). Compound **4** was obtained as white solid (107 mg, 0.063 mmol, 7%). ^{1}H NMR (400 MHz, $CDCl_3$): δ 7.98 (dd, J = 8.3, 2.1 Hz, 10H), 7.35 (dd, J = 8.2, 3.7 Hz, 10H), 6.87-6.83 (m, 10H), 4.59-4.49 (m, 10H), 3.90-3.80 (m, 10H), 3.63-3.52 (m, 10H), 1.92-1.89 (m, 5H), 1.68-1.52 (m, 5H), 1.34-1.29 (m, 5H), 1.12 (d, J = 6.6 Hz, 7.4H), 1.06 (d, J = 6.8 Hz, 7.8H), 0.96 (dt, J = 7.5, 1.6 Hz, 15H). ^{13}C NMR (125 MHz, $CDCl_3$): δ 166.9, 150.8, 150.7, 149.4, 149.3, 143.3, 129.9, 129.6, 128.7, 128.4, 127.2, 127.1, 115.4, 115.2, 73.8, 73.7, 69.8, 52.3, 35.6, 35.5, 29.7, 26.6, 17.2, 16.9, 11.7, 11.6. APCI-HRMS. Calcd for $C_{105}H_{121}O_{20}$ (m/z): $[M + H]^+$,1701.8446. Found: 1701.8404.

Compound 1. To the pillar[5]arene ester **4** (149 mg, 0.088 mmol) in tetrahydrofuran (2.2 mL), sodium hydroxide (105 mg, 2.63 mmol) in water (2.2 mL) was added dropwise. The mixture was refluxed for 24 h. After the organic solvent was removed under reduced pressure, the solution was poured into aqueous HCl solution (1 M, 60 mL). The precipitate was collected by filtration, and washed with water (600 mL). After drying in vacuum, the product was obtained as white solid (137 mg, 0.084 mmol, 95%). ^{1}H NMR (500 MHz, $CDCl_3$): δ 8.02-7.95 (m, 10H), 7.29-7.24 (m, 10H), 6.90-6.80 (m, 10H), 4.73-4.35 (m, 10H), 3.86-3.56 (m, 20H), 1.91-1.24 (m, 30H), 1.13 (t, J = 6.5 Hz, 7.6H), 1.06 (t, J = 7.0 Hz, 7.4 H), 0.98-0.91 (m, 15H). ^{13}C NMR (150 MHz, DMSO-d_6): δ 191.13, 167.07, 149.49, 148.32, 142.13, 130.37, 129.47, 127.82, 127.71, 126.84, 113.99, 113.92, 73.10, 72.92, 68.49, 34.90, 34.71, 28.89, 25.85, 25.75, 16.60, 16.23, 11.23. APCI-HRMS. Calcd for $C_{100}H_{110}O_{20}$ (m/z): $[M + H]^+$, 1631.7663. Found: 1631.7621.

To confirm that the chirality information of **1** observed on CD spectra was ascribed to planar chirality, monomeric compound **10** was synthesized by following the procedure shown in Scheme S2. Synthesis of the precursor **8** was followed literature.[2]

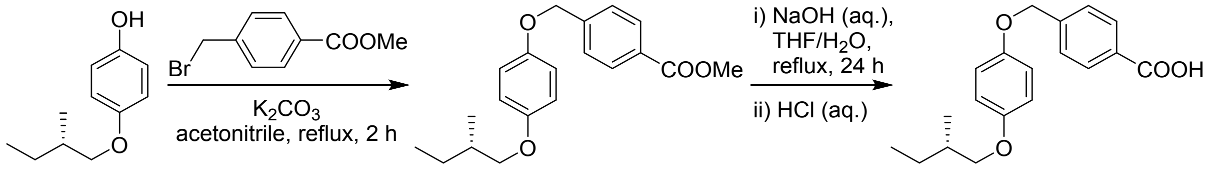

Scheme S2. Synthesis of monomer **10**.

Compound 9. To a stirred solution of mono-substituted dihydroxybenzene **8** (102 mg, 0.57 mmol) in acetonitrile (10 mL) was added methyl 4-(bromomethyl) benzoate (156 mg, 0.68 mmol) and potassium carbonate (94 mg, 0.68 mmol). The reaction mixture was stirred under reflux for 2 h. After cooled down to room temperature, the solid was filtered off. The filtrate was concentrated under reduced pressure. The residue was chromatographed on a silica gel column using a mixture of *n*-hexane and acetone (from 10:1 to 5:1, v/v) as the mobile phase. Compound **9** was obtained as yellow oil (86.0 mg, 0.26 mmol, 48%). ^{1}H NMR (600 MHz,

$CDCl_3$): δ 8.04 (d, J = 8.4 Hz, 2H), 7.49 (d, J = 8.5 Hz, 2H), 6.89-6.81 (m, 4H), 5.07 (s, 2H), 3.92 (s, 3H), 3.76 (dd, J = 9.1, 6.1 Hz, 1H), 3.68 (dd, J = 9.1, 6.6 Hz, 1H), 1.86-1.80 (m, 1H), 1.59-1.52 (m, 1H), 1.28-1.21 (m, 1H), 1.00 (d, J = 6.6 Hz, 3H), 0.94 (t, J = 7.5 Hz, 3H). ^{13}C NMR (100 MHz, $CDCl_3$): δ 167.1, 154.1, 152.6, 142.8, 130.0, 129.7, 127.2, 116.0, 115.6, 73.7, 70.3, 52.3, 34.9, 26.3, 16.7, 11.5. APCI-HRMS. Calcd for $C_{20}H_{24}O_4$ (m/z): $[M + CH_2 + H]^+$, 343.1904. Found: 343.1910.

Compound 10. To a stirred solution of di-substituted dihydroxybenzene **9** (80 mg, 0.244 mmol) in tetrahydrofuran (5 mL), sodium hydroxide (8 mg, 0.300 mmol) in water (5 mL) was added dropwise. The mixture was refluxed for 24 h. After the solvent was removed under reduced pressure, the solution was poured into aqueous HCl solution (1 M, 5 mL). The precipitate was collected by filtration, and washed with water (50 mL). After drying in vacuum, the product was obtained as white solid (67 mg, 0.212 mmol, 88%). ^1H NMR (400 MHz, $CDCl_3$): δ 8.06 (d, J = 8.2 Hz, 2H), 7.50 (d, J = 8.1 Hz, 2H), 6.90-6.82 (m, 4H), 5.08 (s, 2H), 3.79-3.66 (m, 2H), 1.89-1.79 (m, 1H), 1.61-1.51 (m, 1H), 1.30-1.19 (m, 1H), 1.00 (d, J = 6.8 Hz, 3H), 0.94 (t, J = 7.4 Hz, 3H). ^{13}C NMR (125 MHz, $CDCl_3$): δ 171.1, 154.1, 152.6, 143.8, 130.7, 128.7, 127.2, 116.0, 115.6, 73.7, 70.2, 35.0, 26.3, 16.7, 11.5. APCI-HRMS. Calcd for $C_{19}H_{22}O_4$ (m/z): $[M + H]^+$, 315.1591. Found: 315.1592.

CD silencing of 1

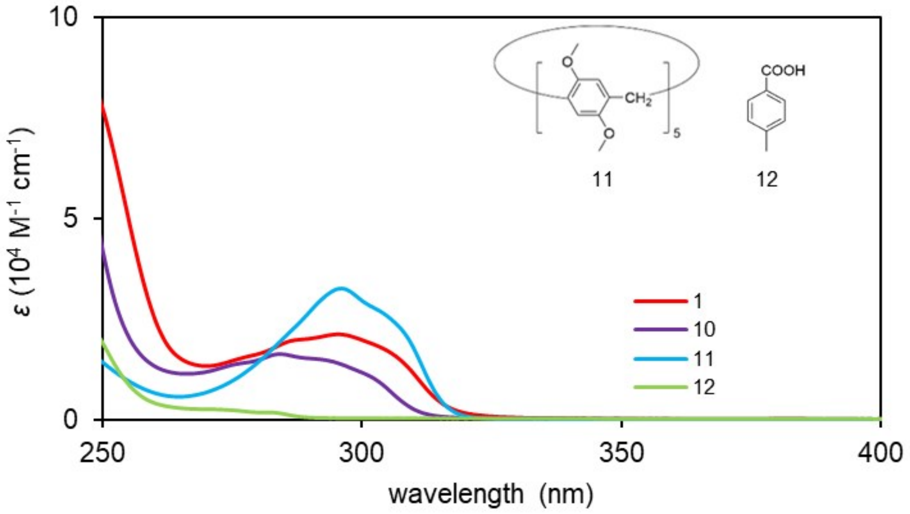

Fig. S1 UV spectra of **1** (0.1 mM), **10** (0.5 mM), **11** (0.1 mM), and **12** (0.125 mM) in chloroform at 20 °C.

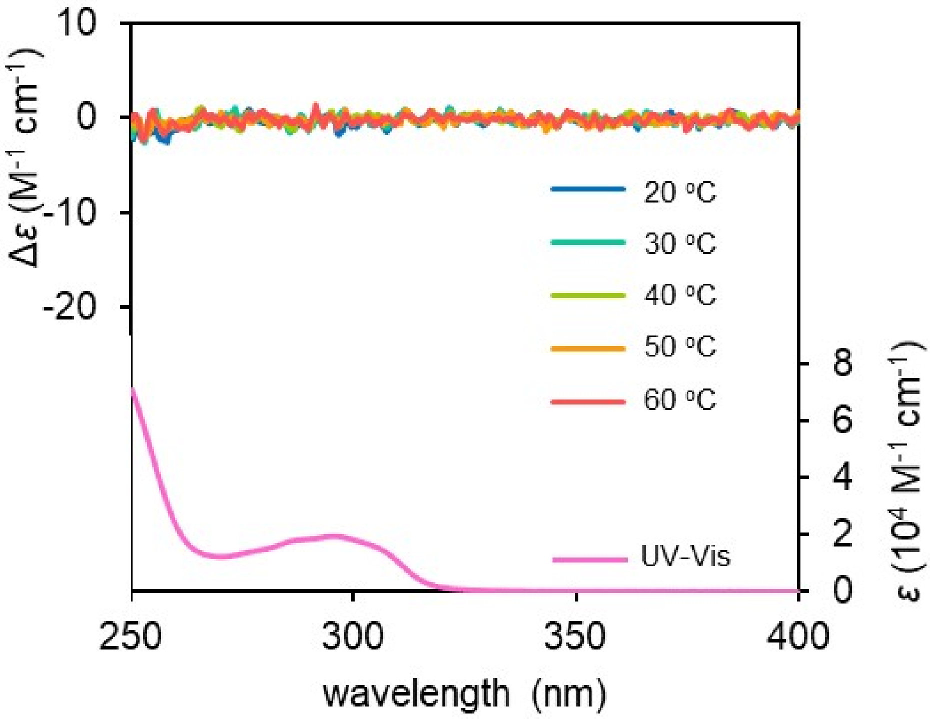

Fig. S2 CD and UV spectra of **1** (0.1 mM) upon heating in chloroform.

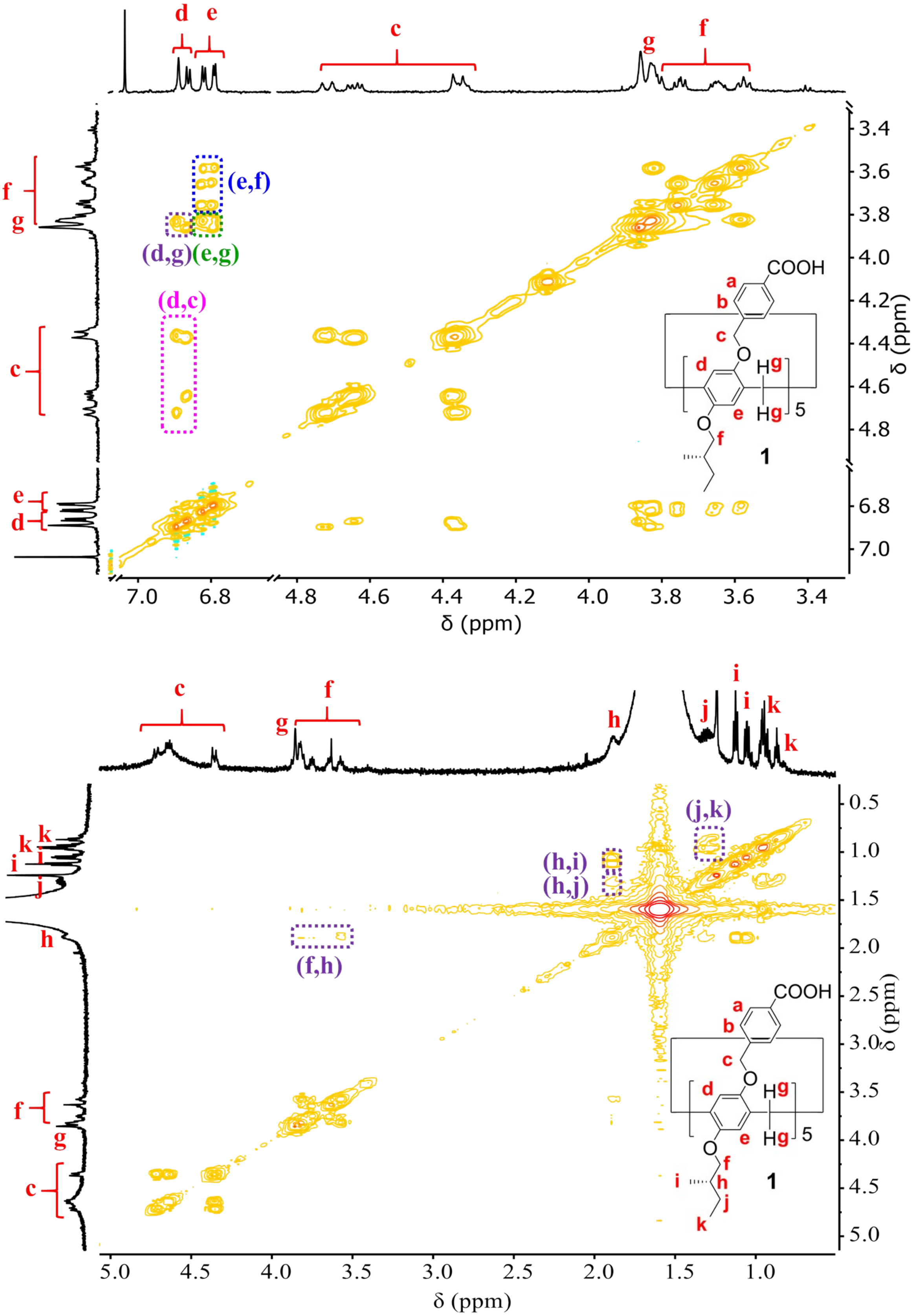

Fig. S3 Partial 2D NOESY NMR spectrum (600 MHz, $CDCl_3$, 303 K, top) and 2D COSY NMR spectrum (600 MHz, $CDCl_3$, 303 K, bottom) of **1** (0.1 mM).

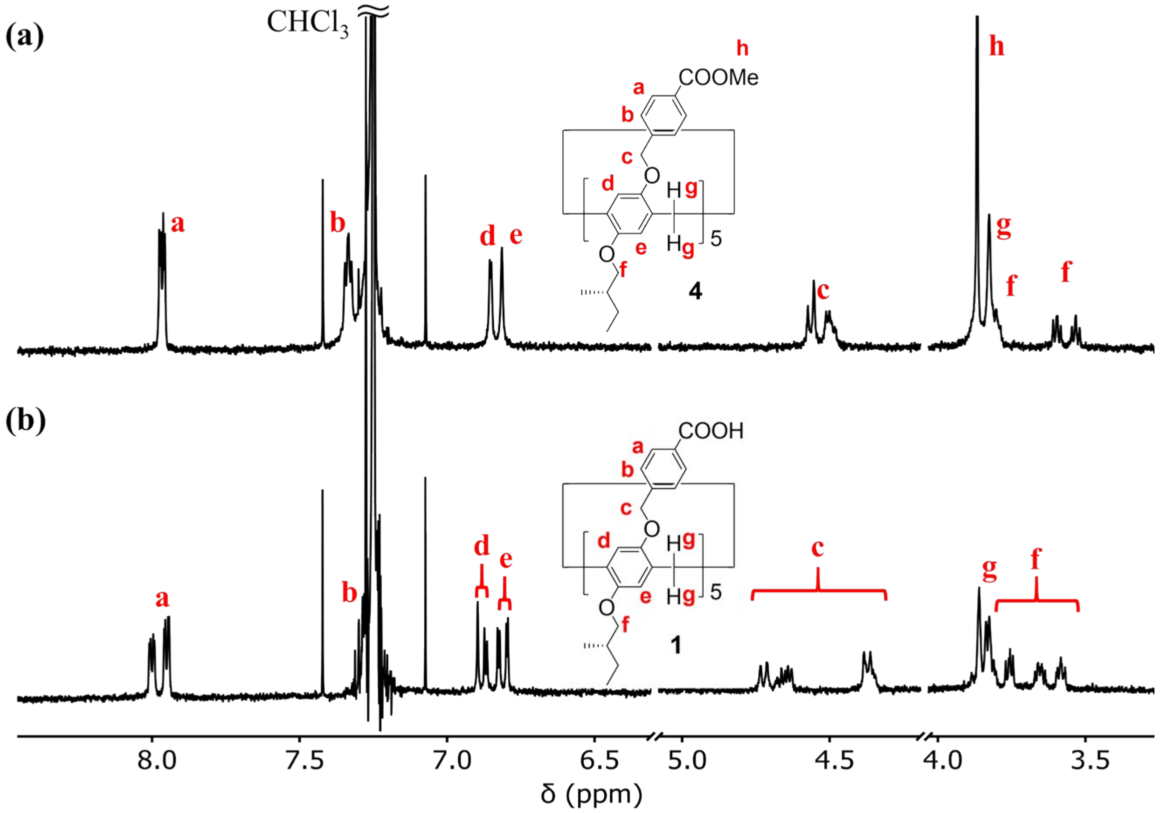

Fig. S4 Partial ^{1}H NMR spectra (600 MHz, $CDCl_3$) of (a) **4** (0.1 mM) and (b) **1** (0.1 mM) at 20 °C. Rim-differentiated pillar[5]arene **4** possesses five asymmetric carbon centers on rims, so there are two sets of peaks on ^{1}H NMR spectrum, which are ascribed to the two diastereomers (*S*,*S*,*S*,*S*,*S*, *pS*,*pS*,*pS*,*pS*,*pS*)- and (*S*,*S*,*S*,*S*,*S*, *pR*,*pR*,*pR*,*pR*,*pR*)-**4** (Fig. S3a, (*pS*)- and (*pR*)- **4** in short). By contrast, four sets of resonance appear on ^{1}H NMR spectrum of **1**. In especial, there are eight peaks altogether in the region of H_d and H_e. Compared with **4**, the molecular structure of **1** has five benzoic acid groups on one rim, which tend to form intramolecular HBN mediated by water molecules,[2] resulting in slow rotation of the units of **1** on NMR time scale.

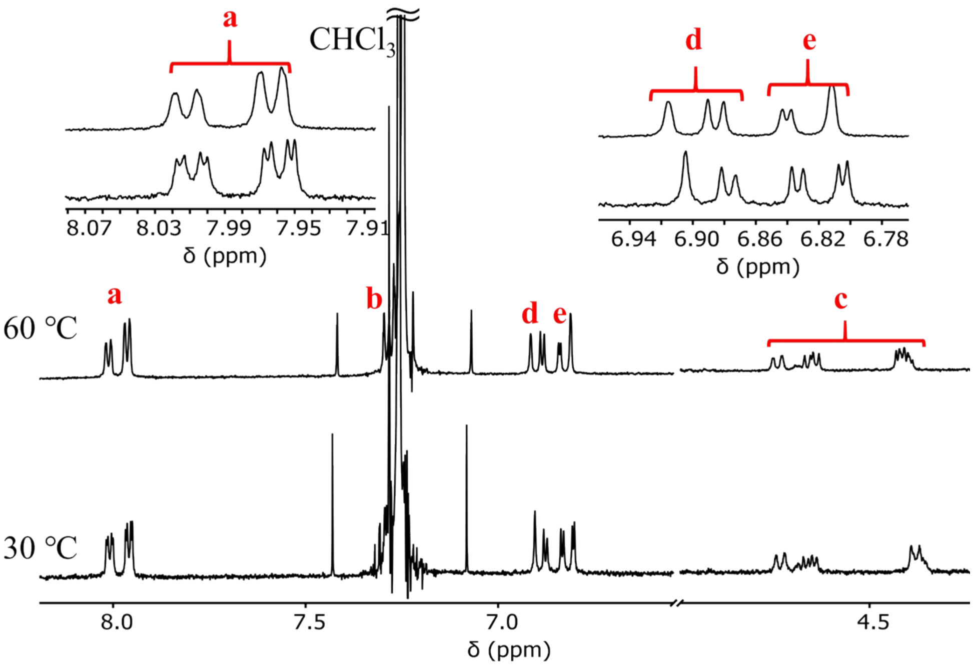

Fig. S5 Partial ^{1}H NMR spectra (600 MHz, $CDCl_3$) of **1** recorded at various temperatures. Increasing the temperature from 30 °C to 60 °C, slight coalescence was observed.

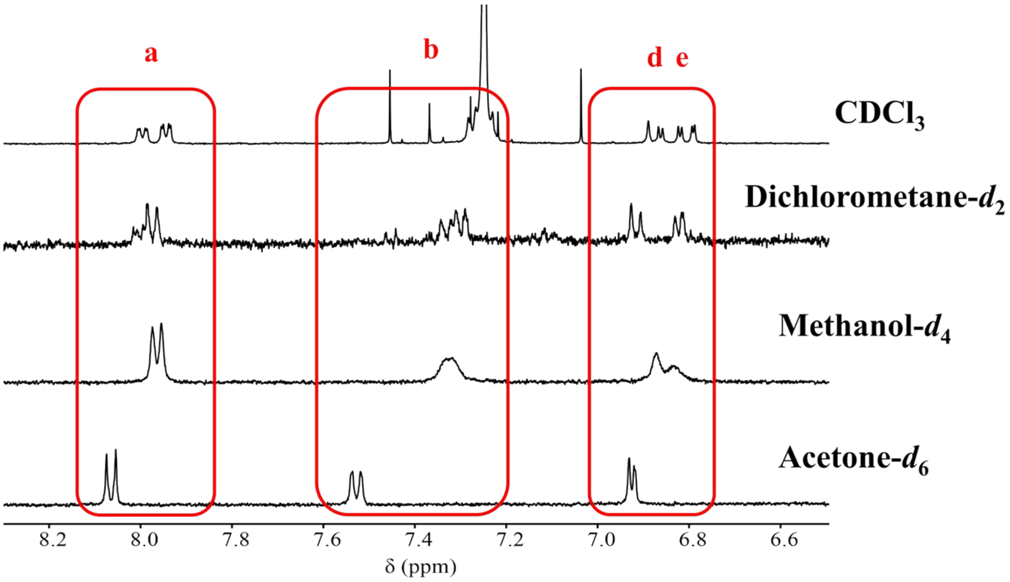

Fig. S6 Partial ^{1}H NMR spectra of **1** (0.1 mM, 400 MHz) in various solvents. In polar solvents (e.g., methanol-d_4 and acetone-d_6), only one set of peaks was observed, indicating that the free rotation of the units was fast on NMR time scale. The HBN was completely broken. In nonpolar solvent (i.e., dichloromethane-d_2), the ^{1}H NMR spectrum was similar to that in $CDCl_3$, due to the slow unit rotation.

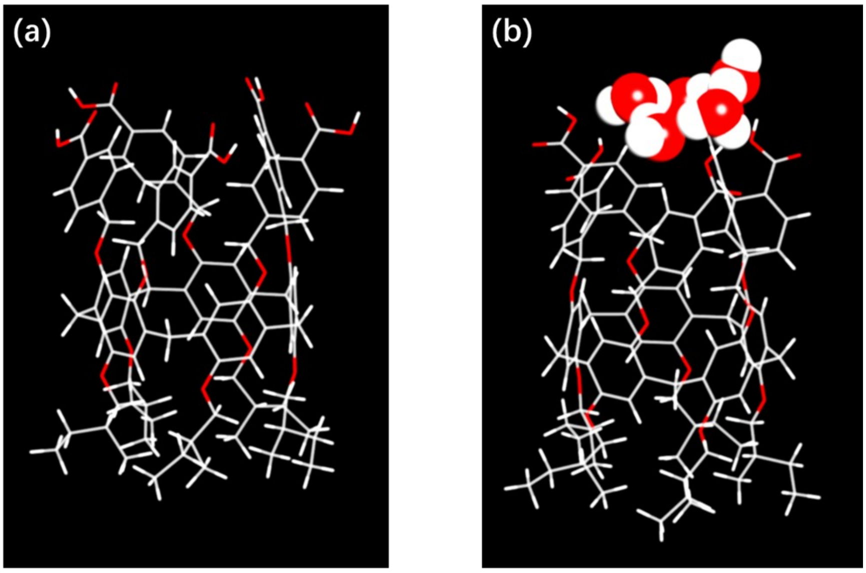

ΔE (kJ/mol) **0** **-53.83**

Fig. S7 Geometrical structures and intermolecular interactions of (a) *pS*-**1** without HBN and (b) *pS*-**1** with HBN mediated via five water molecules obtained from DFT (ωB97XD/6-31G(d,p)) optimization.

CD awakening of 1

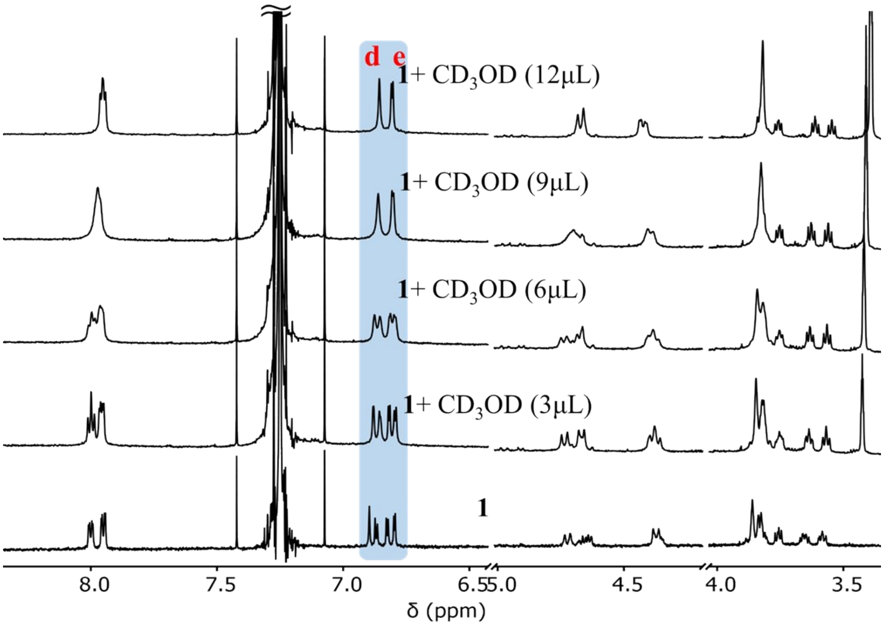

Fig. S8 Partial ^{1}H NMR spectra (600 MHz, $CDCl_3$) of **1** (0.1 mM) upon addition of methanol-d_4. The total volume of $CDCl_3$ was 500 μL in all cases. Upon addition of methanol-d_4, the four sets of resonance coalesced gradually, because methanol is efficient to cut the HBN by forming hydrogen bonds with benzoic acid groups (i.e., solvation).[2] When *ca.* 2% of methanol-d_4 was added, the four sets of peaks coalesced to one set of broad peaks, indicating that the free rotation of the units in **1** was initiated.

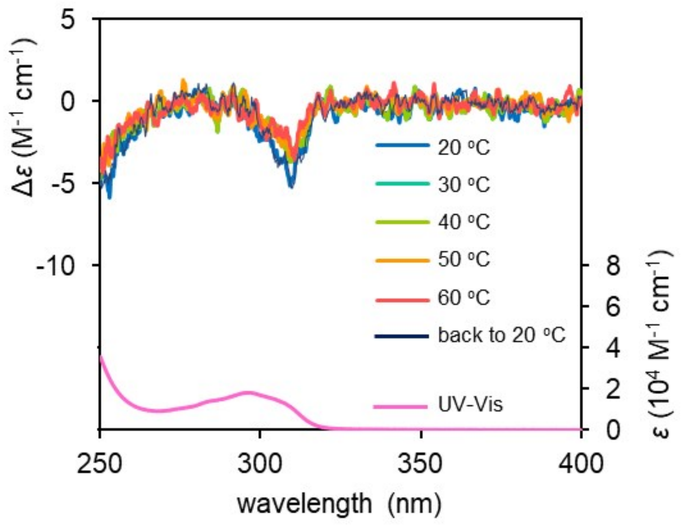

Fig. S9 CD and UV spectra of **1** (0.1 mM) upon heating in chloroform in the presence of 10% of methanol.

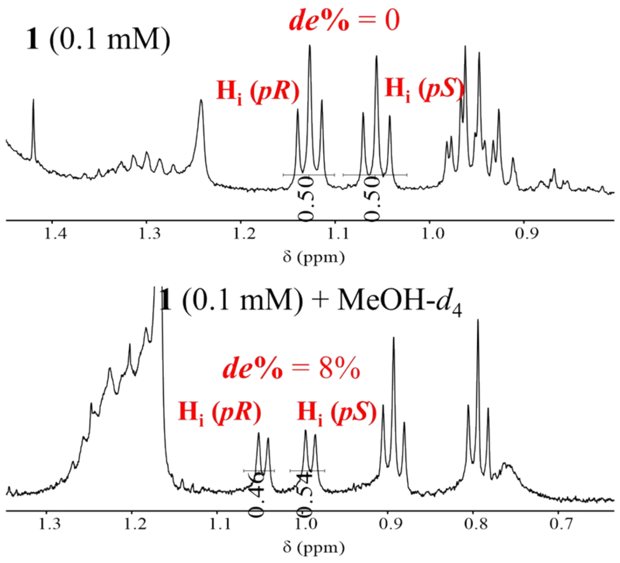

Fig. S10 Partial ^{1}H NMR spectra of **1** (0.1 mM, 400 MHz) without (top) and with (bottom) 10% of methanol-d_4. The diastereomeric excesses were obtained by integrating the *pS*- and *pR*-forms of proton signals of H_i.

Chirality regulation of 1 by DBB and DCE

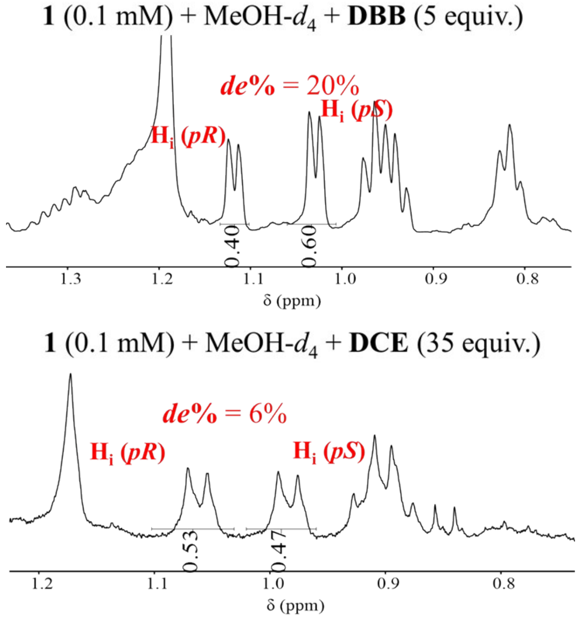

Fig. S11 Partial ^{1}H NMR spectra of **1** (0.1 mM, 400 MHz) with methanol-d_4 (10% of the volume fraction) and **DBB** (5 equiv., top) and **DCE** (35 equiv., bottom). The diastereomeric excesses were obtained by integrating the *pS*- and *pR*-forms of proton signals of H_i.

Discussion on complexation of 1 with DBB and DCE (Fig. S12).

We tried to cultivate single crystals of the complexes between **1** with **DBB** and **DCE** to understand the interaction details in these complexes. Unfortunately, we failed to obtain high-quality single crystal available to analyze by X-ray diffraction. However, our recent work (*Chem. Commun.*, 2020, **56**, 8424−8427),[4] in which we have observed the achiral solvent-dependent CD-signal change of planar chiral pillar[5]arene **13** (Fig. S12), can help us to understand the mechanism of the planar chiral further induction and inversion of **1**.

Pillar[5]arene **13** has the same chiral substituents as **1** on two rims, and showed positive and negative Cotton effects in **DCE** and **DBB**, respectively (*Chem. Commun.*, 2020, **56**, 8424−8427).[4] These observations are same as that in our current research. In our current research, addition of **DCE** in the CD-awakened **1** resulted in positive CD signal of **1**, while addition of **DBB** in CD-awakened **1** caused further negative CD signal (Fig. 3 in the main text).

In Fig. S12, we showed the single crystalline structures of the 1:1 host-guest complex of **13** and **DBB**, and **13** and **DCE**.

In the complex of **13** and **DBB**, the **DBB** molecule is long enough to repel the third carbon atom (i.e., carbons γ and ε) on each substituent (Figure S12c). Thus, the bulky ethyl branch (carbons γ and δ in cyan color) on aliphatic chains are located out of the cavity to minimize the steric hindrance between the bromine atoms of **DBB** and aliphatic chains, which resulted in a tendency of *pS* induction of **13**. Thus, the complex of **13** and **DBB** in crystalline state only shows *pS* chirality. In other words, the (*S*,*S*,*S*,*S*,*S*,*pS*,*pS*,*pS*,*pS*,*pS*)-form of **13** are more stable

than (*S*,*S*,*S*,*S*,*S*,*pR*,*pR*,*pR*,*pR*,*pR*)-form when complexing with **DBB**. Because of the same chiral substituents in **1** and **13**, we believe that complexation of **DBB** and **1** also leads a *pS*-favoring rotation of **1**.

In contrast, with short length, **DCE** is fully buried into the central part of the pillar[5]arene's cavity without interrupting the substituents on rims (Figure S12d). As a consequence, enough space is made in the cavity for the ethyl branch (carbons γ and δ in cyan color) on aliphatic chains, leading a (*S*,*S*,*S*,*S*,*S*,*pR*,*pR*,*pR*,*pR*,*pR*)-form of **13**. Similarly, we believe that complexation with **DCE** also leads a *pR*-favoring rotation of **1**, which make the CD conversion of **1**.

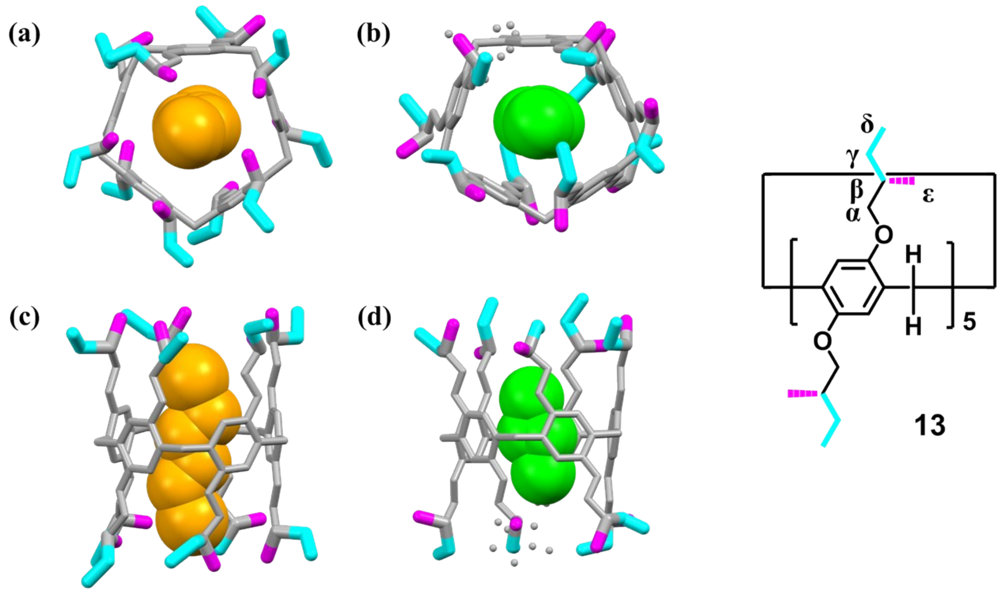

Fig. S12 Single crystal structures of compound **13** complexed with (a,c) **DBB** and (b,d) **DCE**. The two branches of the aliphatic chains on rims of **13** were shown in different colors. Data were taken from ref. 4.

Discussion on chirality regulation of 1 by DBB and DCE in absence of MeOH. Different from the cases of planar chirality awakening **1**, the planar chiral induction effect was very weak when guest molecules were directly added to the solution of **1** in chloroform without awakening the chirality in advance (Fig. 3b). Addition of **DBB** induced *ca*. 30% of the CD intensity of chirality-silenced **1** compared with the chirality-awakened **1**. **DCE** failed to induce the planar chirality of **1** in absence of MeOH.

The weak chiral induction of **1** on addition of **DBB** was caused by the dimerization of **1** as evidenced by the appearance of the dimer peaks on ^{1}H NMR spectra (Fig. S13-S14). To form dimers, the intramolecular HBNs would be broken and rearranged to intermolecular HBNs, which provided a chance for the unit rotation of **1**, thus induced its planar chirality to

some extent. In contrast, **DCE** cannot trigger the dimerization of **1** even in a much excess amount (Fig. S14). The different effect on dimerization of **1** on addition of **DBB** and **DCE** is also ascribed to the length-effect of the guest molecules (Fig. S12). **DCE** may be fully buried into the central part of the pillar[5]arene's cavity without interrupting the intramolecular HBN on rims, while **DBB** is long enough to influence the groups on rims. Nevertheless, awakening the planar chirality of **1** via MeOH in advance was important to launch an effective chiral regulation.

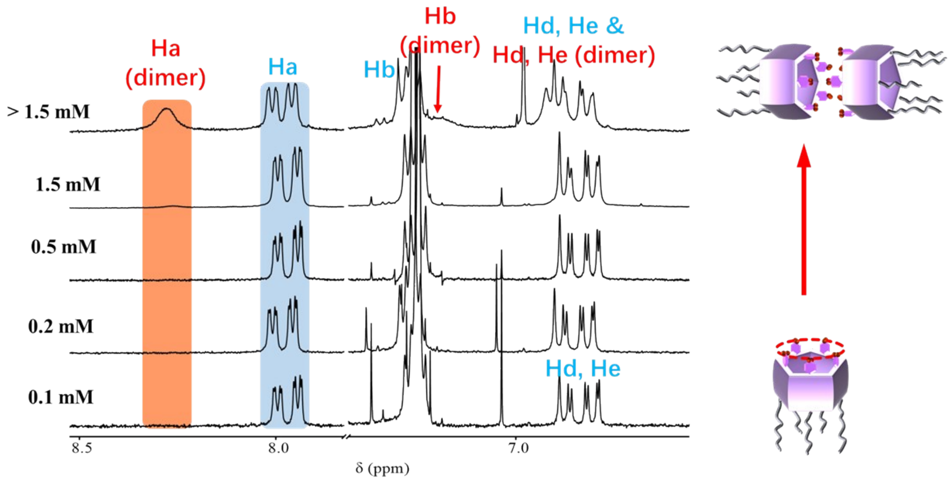

Fig. S13 Partial ^1H NMR spectra (600 MHz, $CDCl_3$) of **1** at various concentrations. At diluted concentrations (0.1–0.5 mM), no change was observed on ^1H NMR spectra. As the concentration increased to 1.5 mM and higher, a new set of peaks emerged (H_a (dimer) and H_b (dimer)), which implied that self-assemblies formed as concentration was increased. Similar behavior has been observed in our previous work,[2] where evidences demonstrated the concentration-dependent dimerization of compounds similar to **1** via intermolecular hydrogen bonds. Therefore, we assigned the emerging peaks as dimers of **1**.

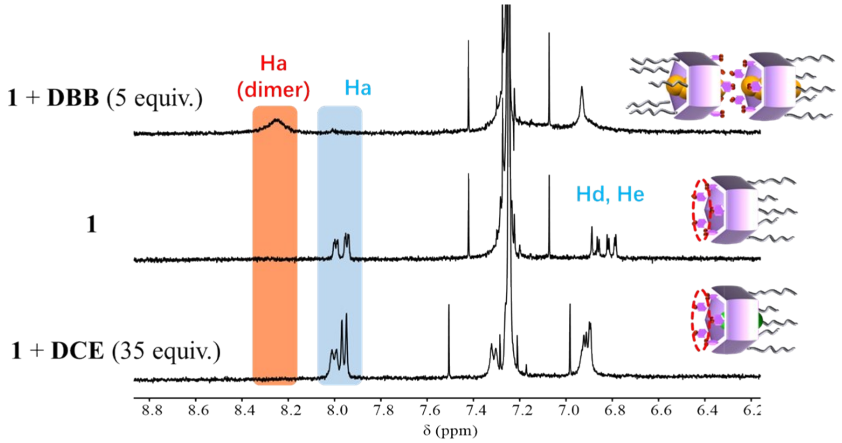

Fig. S14 Partial ^1H NMR spectra of **1** (0.1 mM) with **DBB** (5 equiv.) and **DCE** (35 equiv.) in $CDCl_3$ at 20 °C. Upon addition of 5 equiv. of **DBB** in the solution of **1** (0.1 mM), all monomeric **1** was dimerized. By contrast, even 35 equiv. of **DCE** was not able to trigger the dimerization of **1**. The different effect on dimerization of **1** on addition of **DBB** and **DCE** is ascribed to the length-effect of the guest molecules. As mentioned above (Fig. S12), the **DBB** molecule is long

enough to repel the third carbon atom on each substituent. This will surely interrupt the intramolecular HBN on the other rim of **1**. As a consequence, the stability of the intramolecular HBN decrease, which gives a chance for dimerization of **1** by forming intermolecular hydrogen bonds. In contrast, **DCE** is fully buried into the central part of the pillar[5]arene's cavity without interrupting the intramolecular HBN on rims.

Nanotube formation of 1 and 2

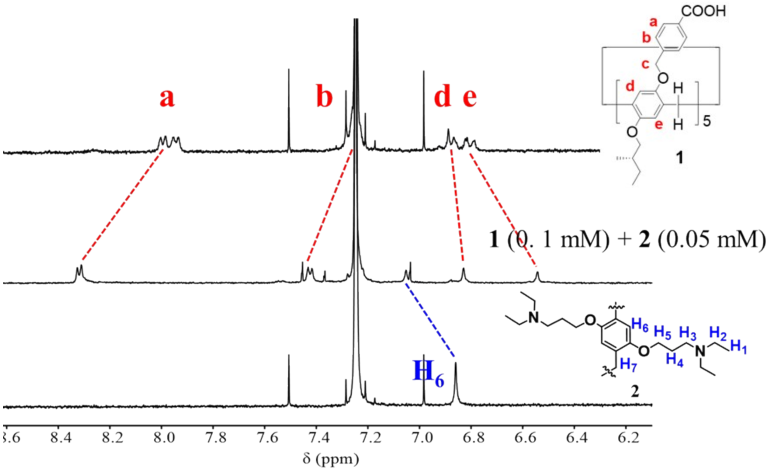

Fig. S15 (a) ^{1}H NMR spectra of **1** (0.1 mM), **2** (0.05 mM) and the trimeric nanotubes via mixing **1** (0.1 mM) and **2** (0.05 mM) in $CDCl_3$. Mixing **1** and **2** with a 1:2 ratio resulted in clear and simple ^{1}H NMR spectrum. This suggested the formation of a single species $\mathbf{1}_2 \cdot \mathbf{2}$ in solution.

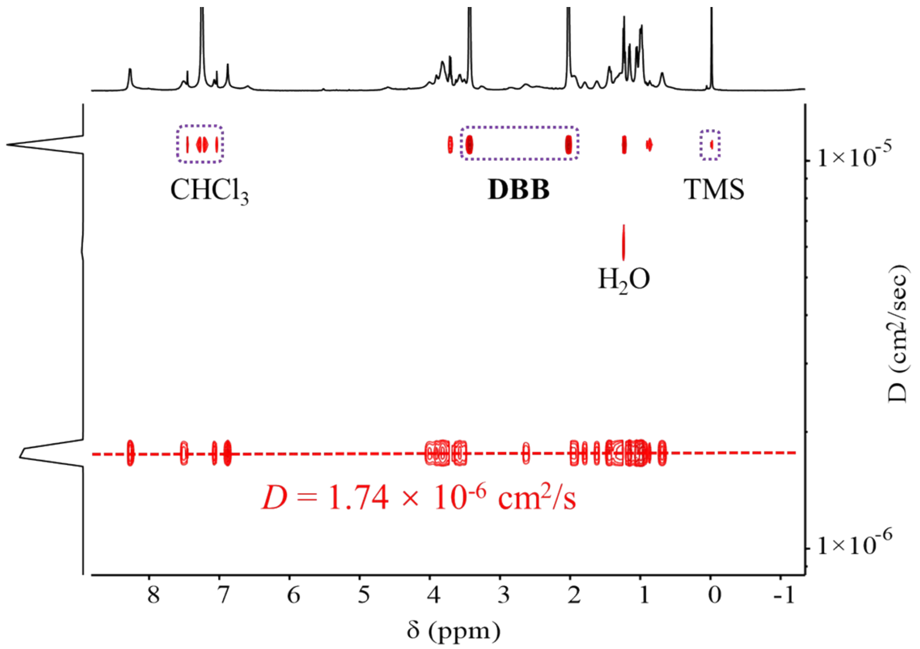

Fig. S16 DOSY spectrum (600 MHz, $CDCl_3$, 30 ℃) of the mixture of **1** (0.1 mM) and **2** (0.05 mM) with **DBB** (0.5 mM). The identical diffusion constants of the peaks on both **1** and **2** indicated that **1** and **2** assembled into a single component in solution, which confirmed the formation of trimeric nanotubes.

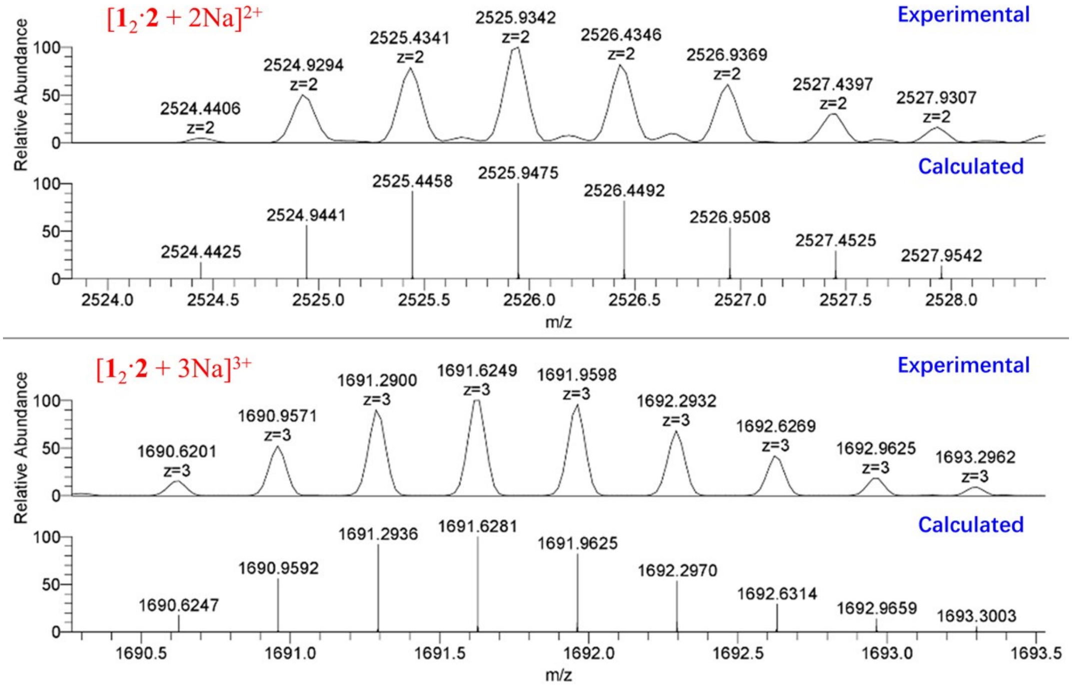

Fig. S17 Experimental and calculated electrospray ionization peaks of the trimeric nanotube ($\mathbf{1}_2{\cdot}\mathbf{2}$).

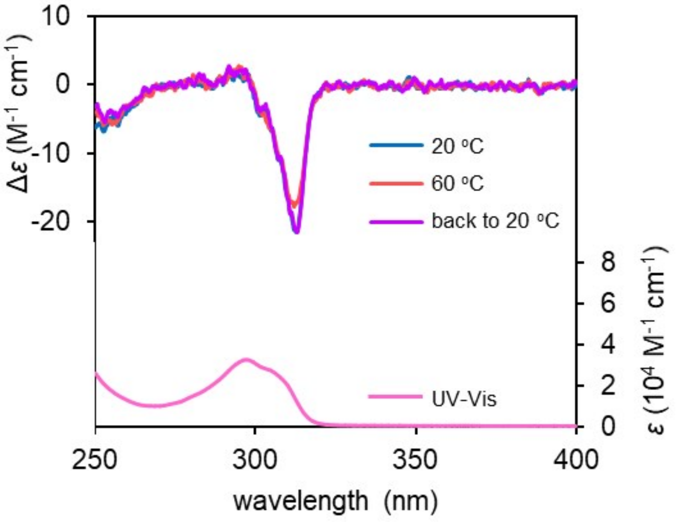

Fig. S18 CD and UV spectra of trimeric nanotubes prepared by pre-regulation strategy (Path A in Fig. 4) upon heating and cooling.

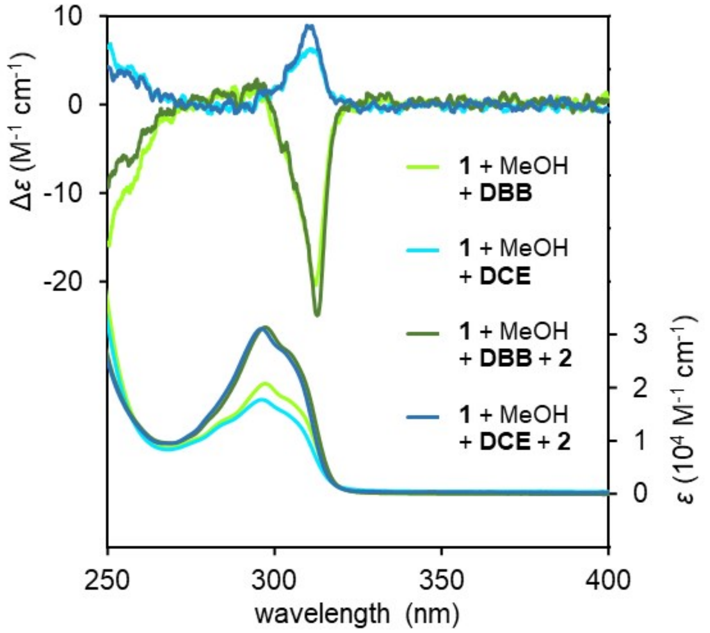

Fig. S19 CD and UV spectra of *pS*- and *pR*-rich trimeric nanotubes prepared by pre-regulation strategy.

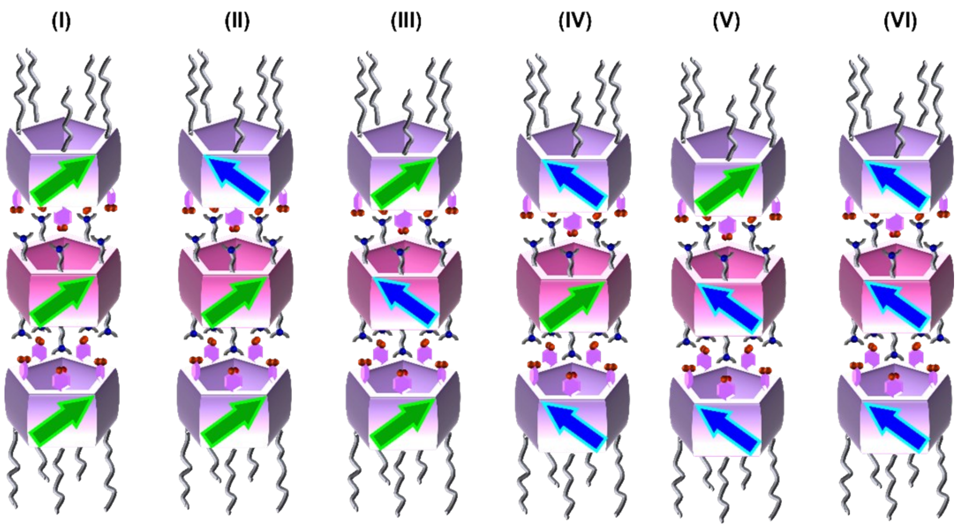

Fig. S20 Possible structures of chiral trimeric nanotubes. There are six possible combinations of **1** and **2** in trimeric nanotubes. However, structures (II), (III), (IV) and (V) would be less stable than (I) and (VI), because steric hindrance between the neighboring pillar[5]arenes with opposite planar chirality may decrease the stability of the trimeric structures as what observed in our previous research.[2] Therefore, upon addition of 0.5 equiv. of **2** in the pre-regulated **1** (i.e., **1** + MeOH + **DBB** and **1** + MeOH + **DCE**), the resulted nanotubes would mainly adopt forms (I) and (VI), respectively.

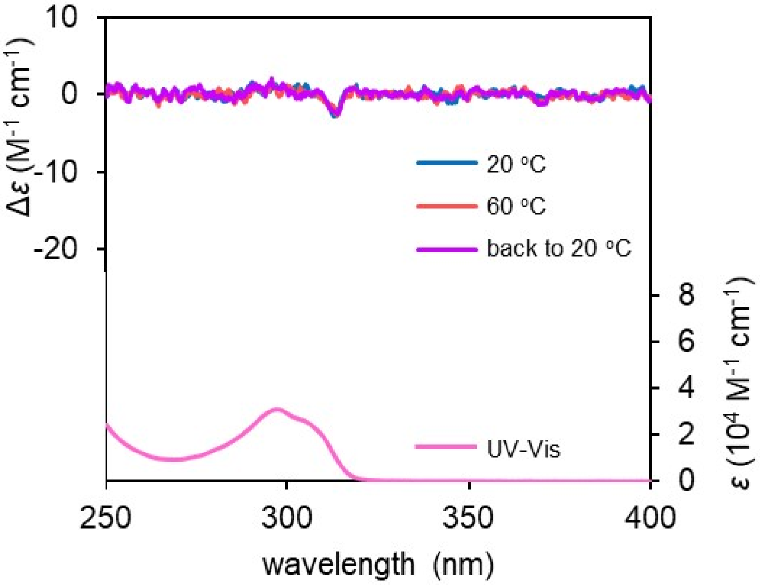

Fig. S21 CD and UV spectra of trimeric nanotubes prepared by post-regulation strategy (Path B in Fig. 4) upon heating and cooling.

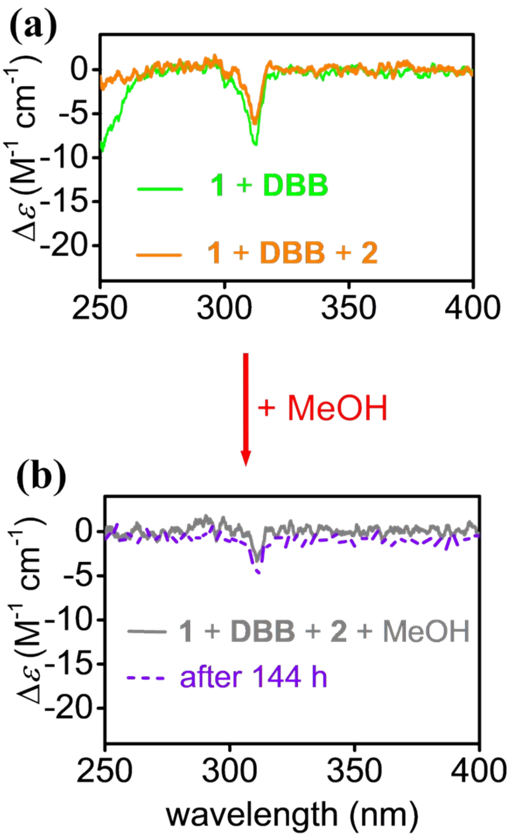

Fig. S22 CD spectra of (a) the trimeric nanotubes prepared from partially regulated **1** (0.1 mM) and (b) upon further adding MeOH (10% volume fraction of the solution) (Path C in Fig. 4).

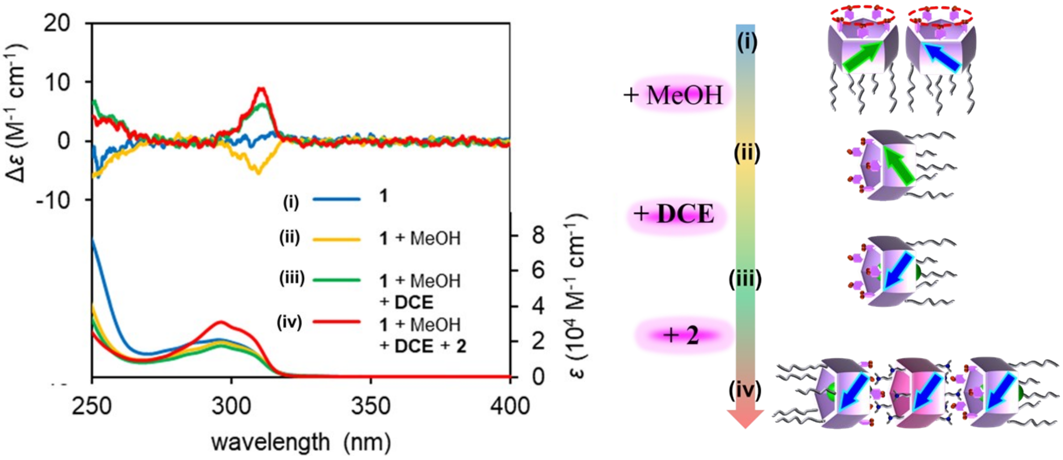

Fig. S23 Pre-regulation of **1** for preparing *pR*-rich nanotubes (Path A in Fig. S26). CD changes of **1** (0.1 mM) upon addition of MeOH (10% volume fraction of the solution), **DCE** (35 equiv.) and **2** (0.5 equiv.) by the illustrated sequence.

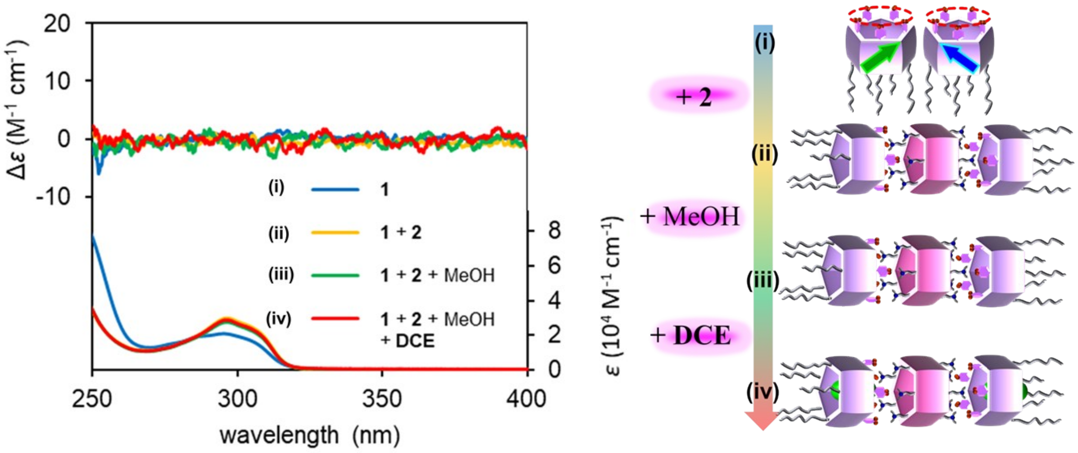

Fig. S24 Path B in Fig. S26. CD changes of **1** (0.1 mM) upon addition of **2** (0.5 equiv.), MeOH (10% volume fraction of the solution) and **DCE** (5 equiv.) by the illustrated sequence.

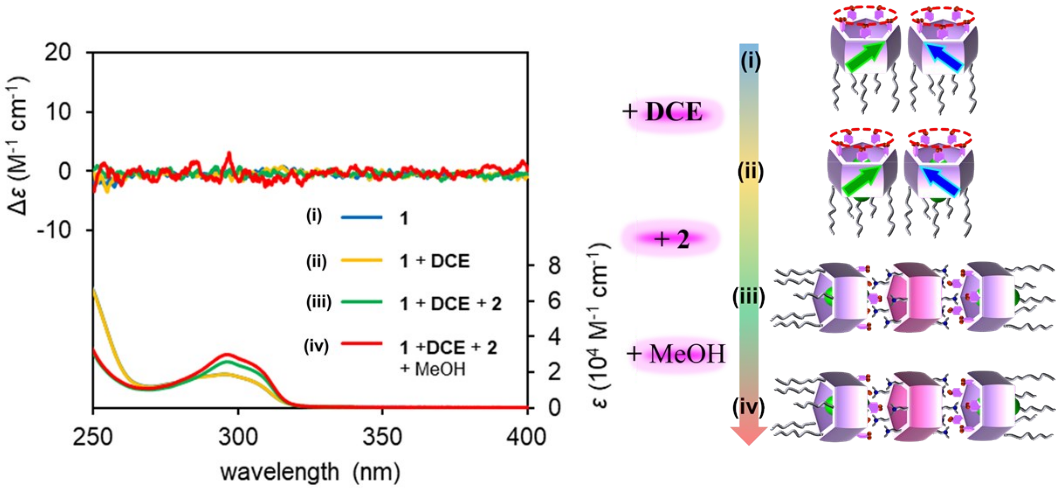

Fig. S25 Path C in Fig. S26. CD changes of **1** (0.1 mM) upon addition of **DCE** (35 equiv.), **2** (0.5 equiv.) and MeOH (10% volume fraction of the solution) by the illustrated sequence.

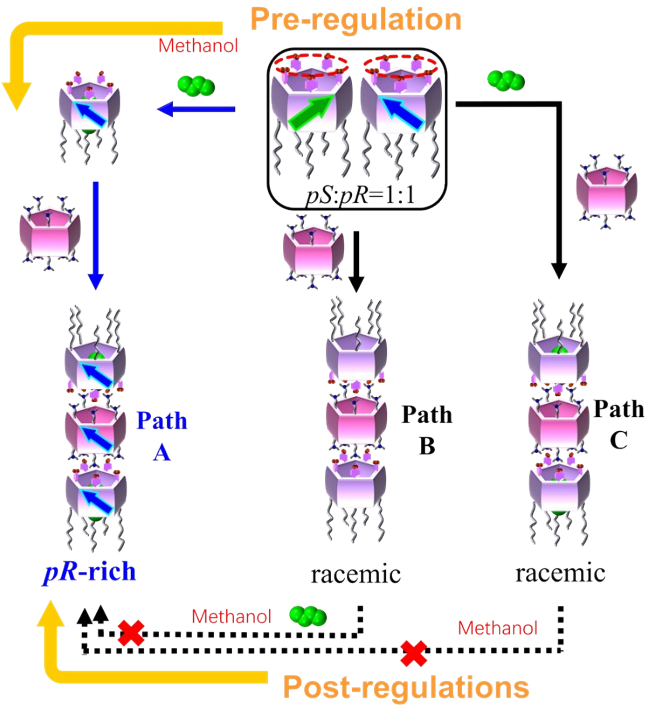

Fig. S26 Illustration of *pR*-rich nanotubes prepared by pre- and post-regulation strategies.

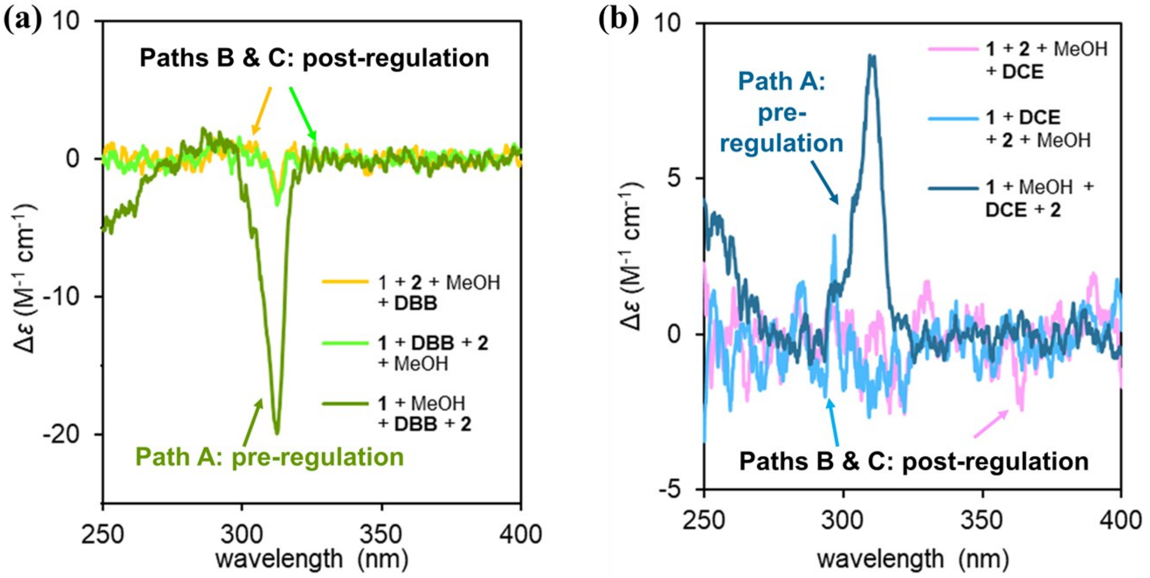

Fig. S27 A summary of the pre- and post-regulation results. (a) CD spectra of *pS*-rich nanotubes constructed from **1** (0.1 mM), **2** (0.5 equiv.), MeOH (10% volume fraction of the solution), and **DBB** (5 equiv.) in different sequences (Fig. 4 and S22). (b) CD spectra of *pR*-rich nanotubes constructed from **1** (0.1 mM), **2** (0.5 equiv.), MeOH (10% volume fraction of the solution), and **DCE** (35 equiv.) in different sequences (Fig. S23-26).

Chirality storage of the nanotubes after removing awakener and regulators

By removing the solvent and heating the pre-regulated nanotubes at 120 ℃ for 5 days in vacuum, we tried to remove all external factors (i.e., methanol, **DBB** and **DCE**) that was used to regulate the chirality of **1**. By analyzing the ^{1}H NMR spectra before and after heating, we found that all the external factors (methanol and **DBB**) in the *pS*-rich nanotubes prepared by path A were removed (Fig. S28). The *pR*-rich nanotubes prepared by pre-regulation show weak CD signals. After removal of **DCE** and methanol, the CD changes were difficult to monitor. Therefore, we only checked the stability of the aforementioned *pS*-rich nanotubes after removing external factors in solution by CD measurement (Fig. S29).

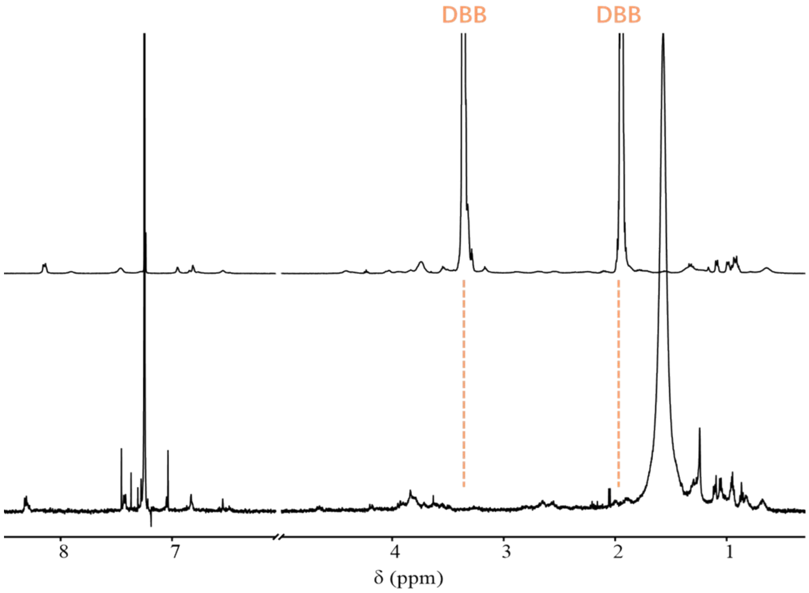

Fig. S28 Partial ^{1}H NMR spectra of *pS*-rich nanotubes (0.05 mM, $CDCl_3$) prepared by path A (top) and after heating the sample in vacuum at 120 ℃ for 5 days (bottom). **DBB** was completely removed as evidenced by the disappearance of the **DBB** proton signals after heating.

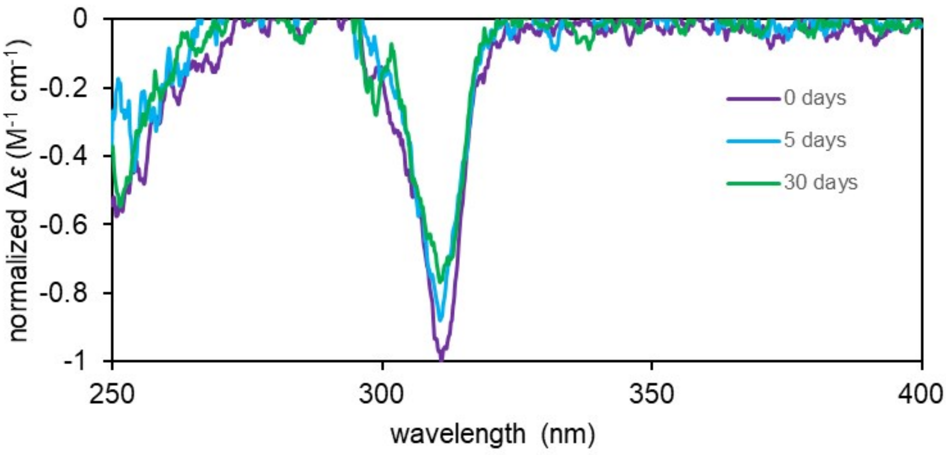

Fig. S29 Time-dependent normalized CD spectra (chloroform, 20 ℃) of the *pS*-rich nanotubes prepared by path A after removing **DBB** and methanol.

Supplementary discussion

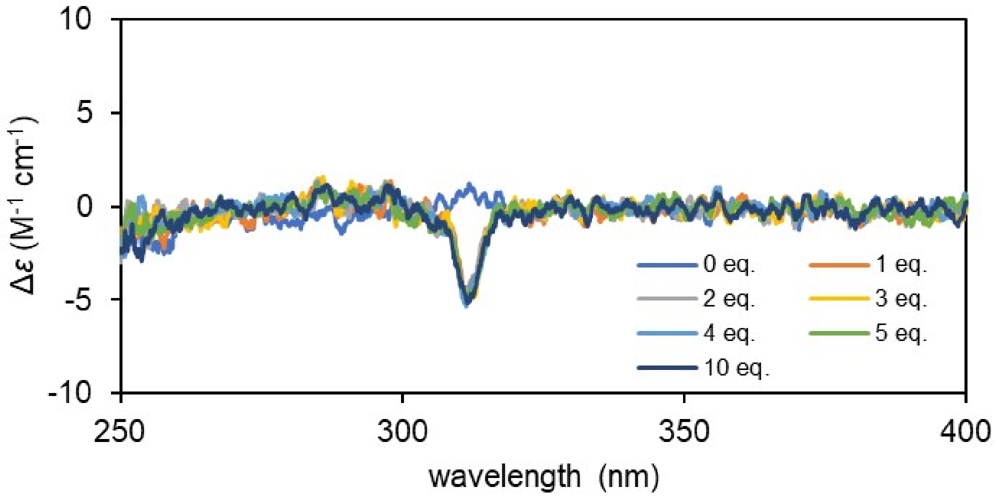

Fig. S30 Optimization of the charge amount of **DBB**. The CD spectra ($CHCl_3$) of **1** (0.1 mM) upon addition of **DBB** (0 - 10 equiv.) were recorded at 20 ℃. The CD intensity slightly changed upon addition of **DBB** from **1** to 5 equiv.; on further addition of **DBB**, the CD changes were negligible. Thus, 5 equiv. of **DBB** was chosen for all other experiments in this research.

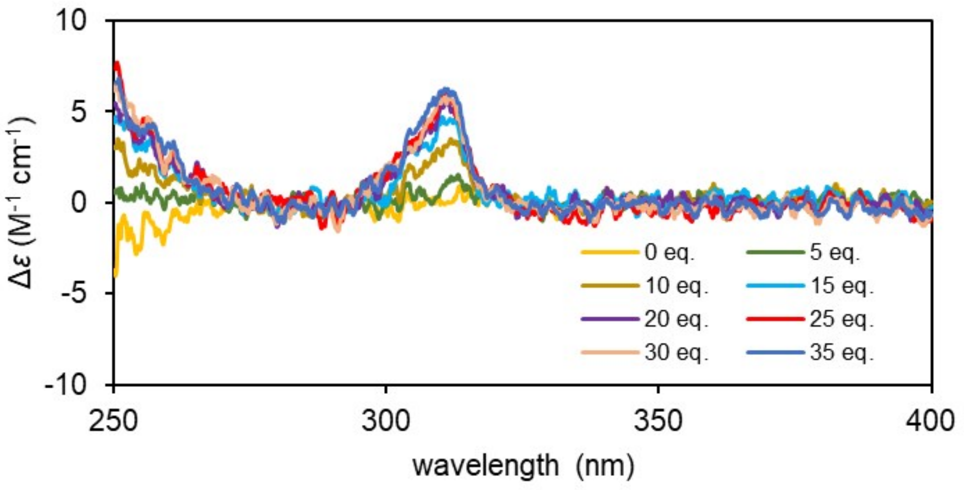

Fig. S31 Optimization of the charge amount of **DCE**. The CD spectra ($CHCl_3$) of **1** (0.1 mM) with MeOH (10% volume fraction of the solution) upon addition of **DCE** (0 - 35 equiv.) were recorded at 20 ℃. Upon addition of **DCE**, the CD intensity gradually increased until 35 equiv. of **DCE** was added. Thus, 35 equiv. of **DCE** was chosen for all other experiments in this research.

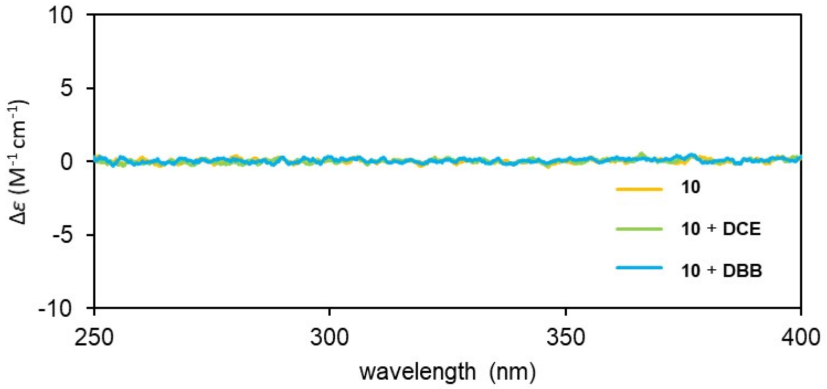

Fig. S32 CD spectra ($CHCl_3$) of **10** (0.1 mM, orange line) upon addition of 25 equiv. of **DBB** (blue line) and 125 equiv. of **DCE** (green line). No CD signals were observed upon addition of linear guests. It is clear that the macrocyclic structure was essential for chiral induction upon addition of linear guests.

References

1. Gaussian 16, Revision C.01 M. J. Frisch, G. W. Trucks, H. B. Schlegel, G. E. Scuseria, M. A. Robb, J. R. Cheeseman, G. Scalmani, V. Barone, G. A. Petersson, H. Nakatsuji, X. Li, M. Caricato, A. V. Marenich, J. Bloino, B. G. Janesko, R. Gomperts, B. Mennucci, H. P. Hratchian, J. V. Ortiz, A. F. Izmaylov, J. L. Sonnenberg, D. Williams-Young, F. Ding, F. Lipparini, F. Egidi, J. Goings, B. Peng, A. Petrone, T. Henderson, D. Ranasinghe, V. G. Zakrzewski, J. Gao, N. Rega, G. Zheng, W. Liang, M. Hada, M. Ehara, K. Toyota, R. Fukuda, J. Hasegawa, M. Ishida, T. Nakajima, Y. Honda, O. Kitao, H. Nakai, T. Vreven, K. Throssell, J. A. Montgomery, Jr., J. E. Peralta, F. Ogliaro, M. J. Bearpark, J. J. Heyd, E. N. Brothers, K. N. Kudin, V. N. Staroverov, T. A. Keith, R. Kobayashi, J. Normand, K. Raghavachari, A. P. Rendell, J. C. Burant, S. S. Iyengar, J. Tomasi, M. Cossi, J. M. Millam, M. Klene, C. Adamo, R. Cammi, J. W. Ochterski, R. L. Martin, K. Morokuma, O. Farkas, J. B. Foresman, and D. J. Fox, Gaussian, Inc., Wallingford CT, 2016.
2. S. Fa, Y. Sakata, S. Akine and T. Ogoshi, *Angew. Chem. Int. Ed.*, 2020, **59**, 9309–9313.
3. K. Mori, T. Tashiro, B. Zhao, D. Suckling and A. El-Sayed, *Tetrahedron*, 2010, **6**, 2642–2653.
4. Y. Nagata, M. Suzuki, Y. Shimada, H. Sengoku, S. Nishida, T. Kakuta, T. Yamagishi, M. Suginome and T. Ogoshi, *Chem. Commun.*, 2020, **56**, 8424−8427.

^{1}H & ^{13}C NMR Spectra

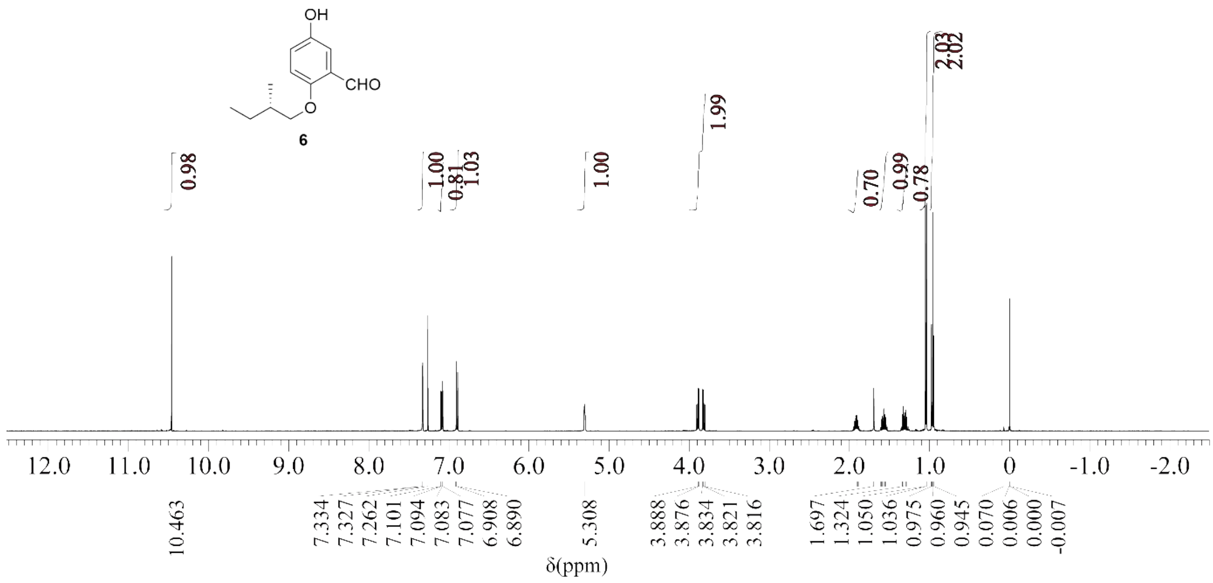

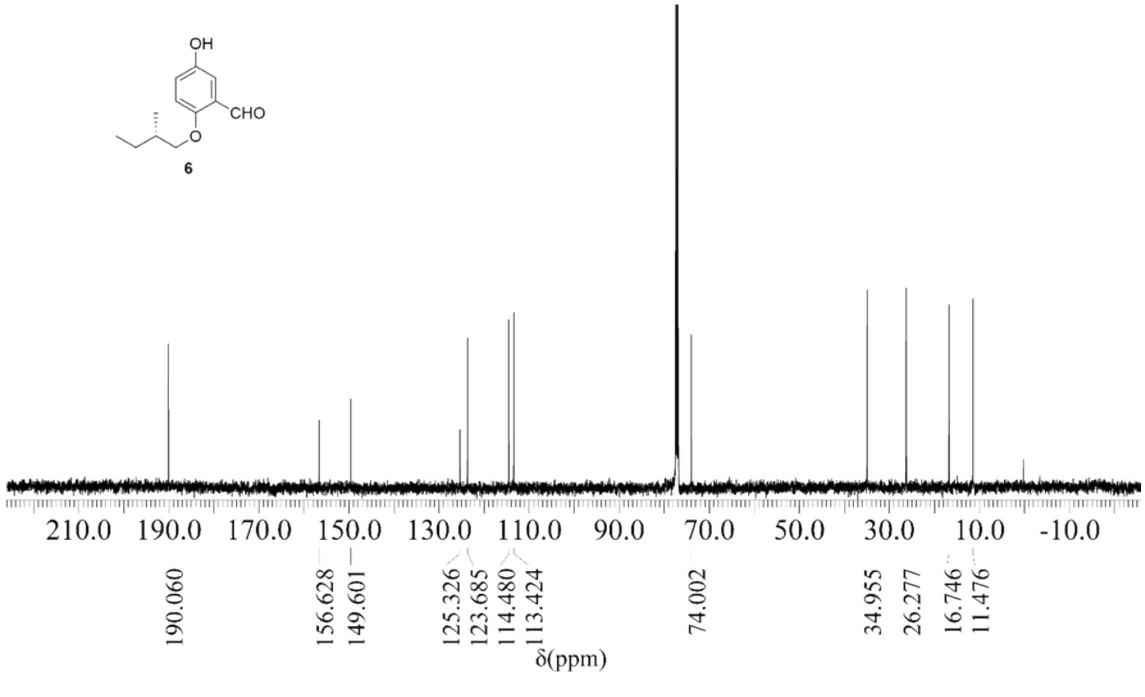

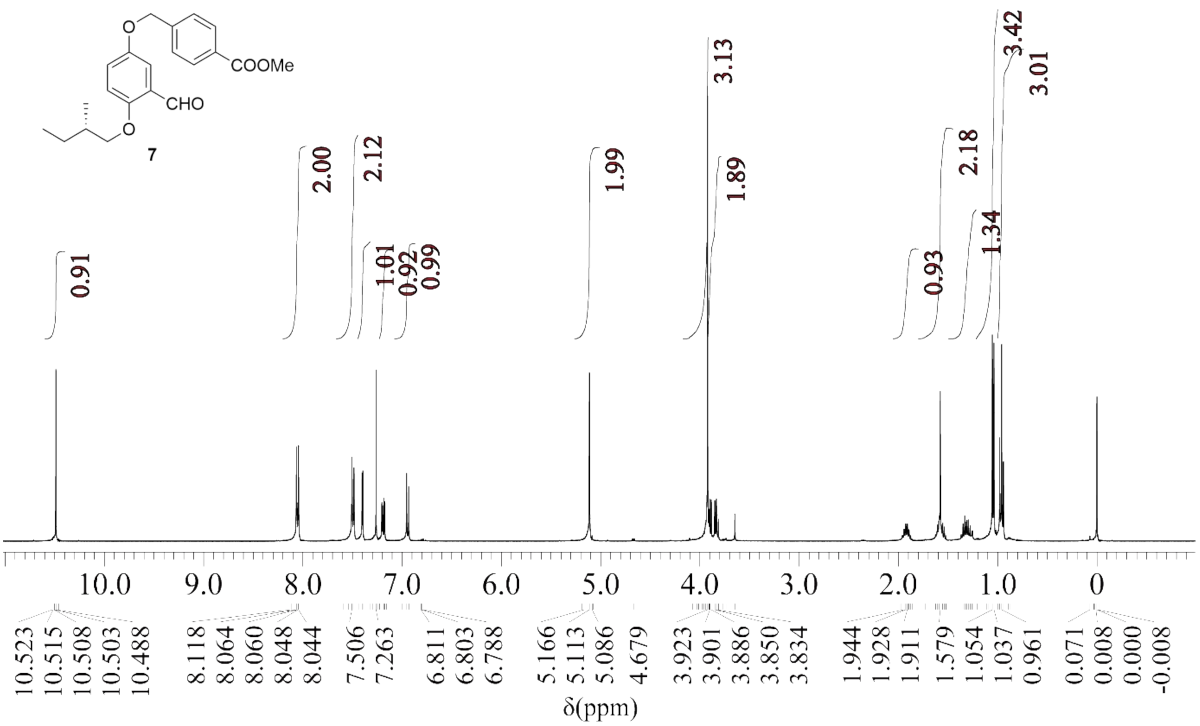
COOMe
CHO
7
0.91
2.00
2.12
1.01
0.92
0.99
1.99
3.13
1.89
0.93
2.18
1.34
3.42
3.01
10.0 9.0 8.0 7.0 6.0 5.0 4.0 3.0 2.0 1.0 0
10.523 10.515 10.508 10.503 10.488
8.118 8.064 8.060 8.048 8.044
7.506 7.263
6.811 6.803 6.788
5.166 5.113 5.086 4.679
3.923 3.901 3.886 3.850 3.834
1.944 1.928 1.911 1.579 1.054 1.037 0.961
0.071 0.008 0.000 -0.008
δ(ppm)

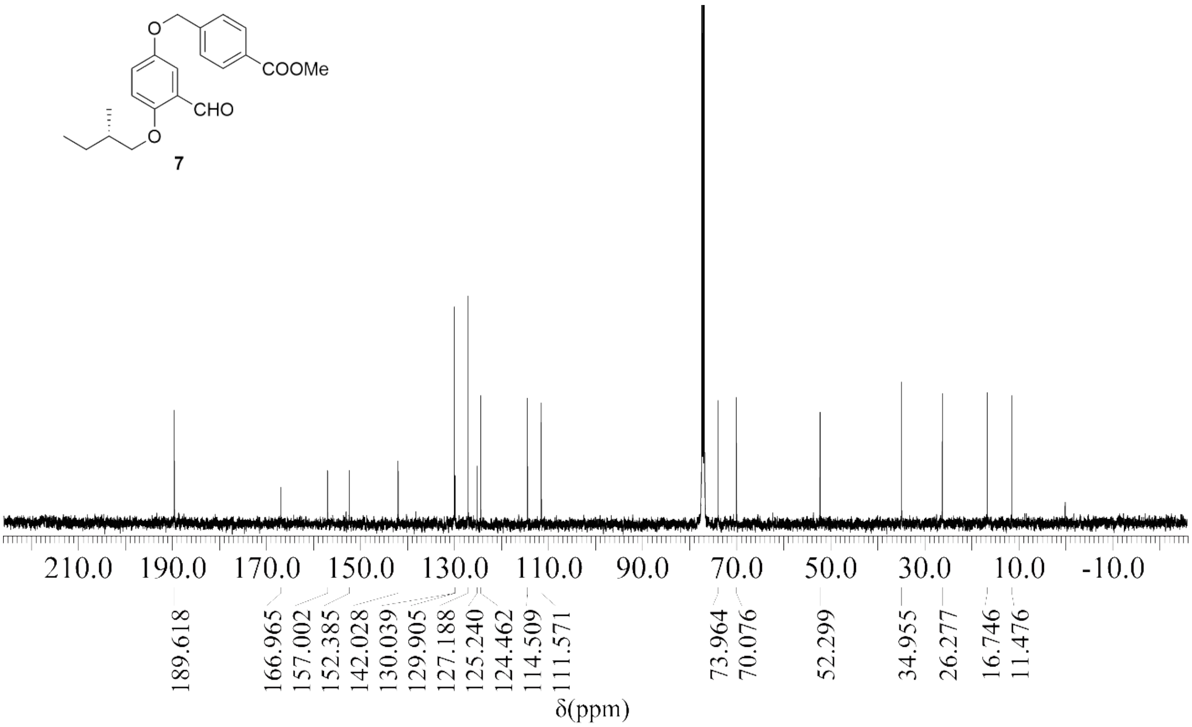
COOMe
CHO
7
210.0 190.0 170.0 150.0 130.0 110.0 90.0 70.0 50.0 30.0 10.0 -10.0
189.618
166.965 157.002 152.385 142.028 130.039 129.905 127.188 125.240 124.462 114.509 111.571
73.964 70.076
52.299
34.955 26.277 16.746 11.476
δ(ppm)

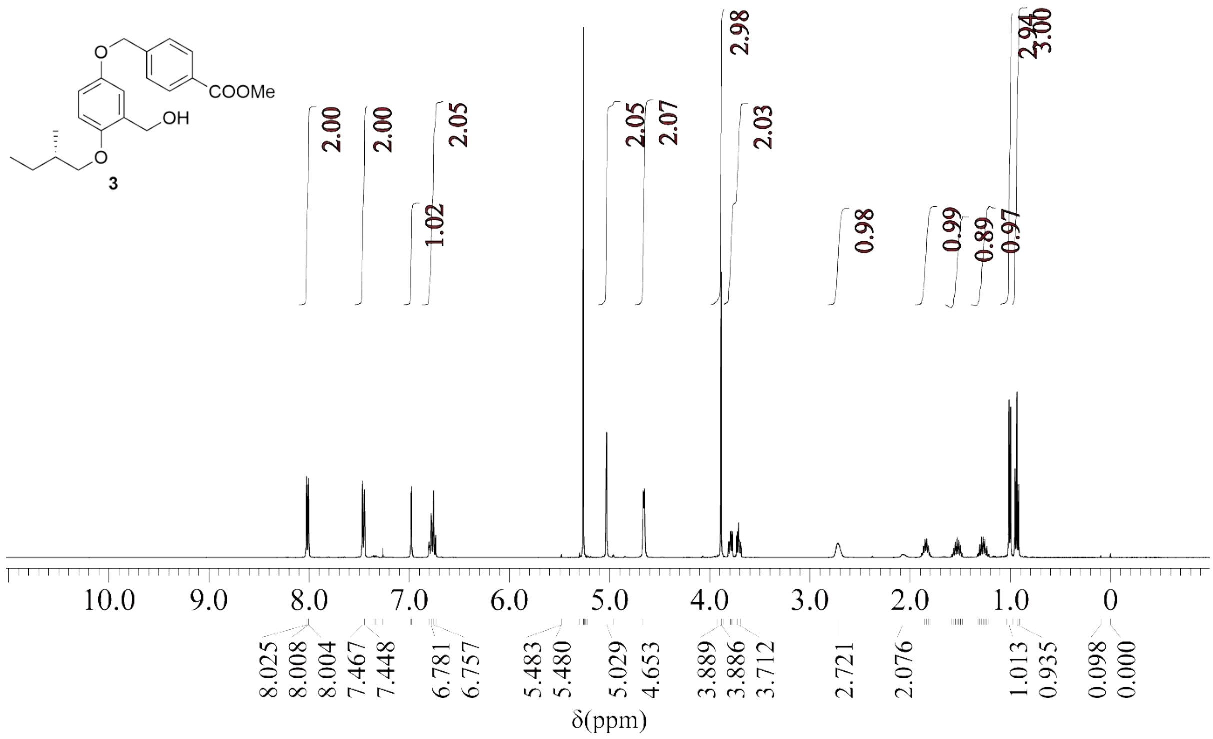
COOMe
OH
3
2.98
2.94
3.00
2.00
2.00
2.05
2.05
2.07
2.03
1.02
0.98
0.99
0.89
0.97
10.0 9.0 8.0 7.0 6.0 5.0 4.0 3.0 2.0 1.0 0
8.025
8.008
8.004
7.467
7.448
6.781
6.757
5.483
5.480
5.029
4.653
3.889
3.886
3.712
2.721
2.076
1.013
0.935
0.098
0.000
δ(ppm)

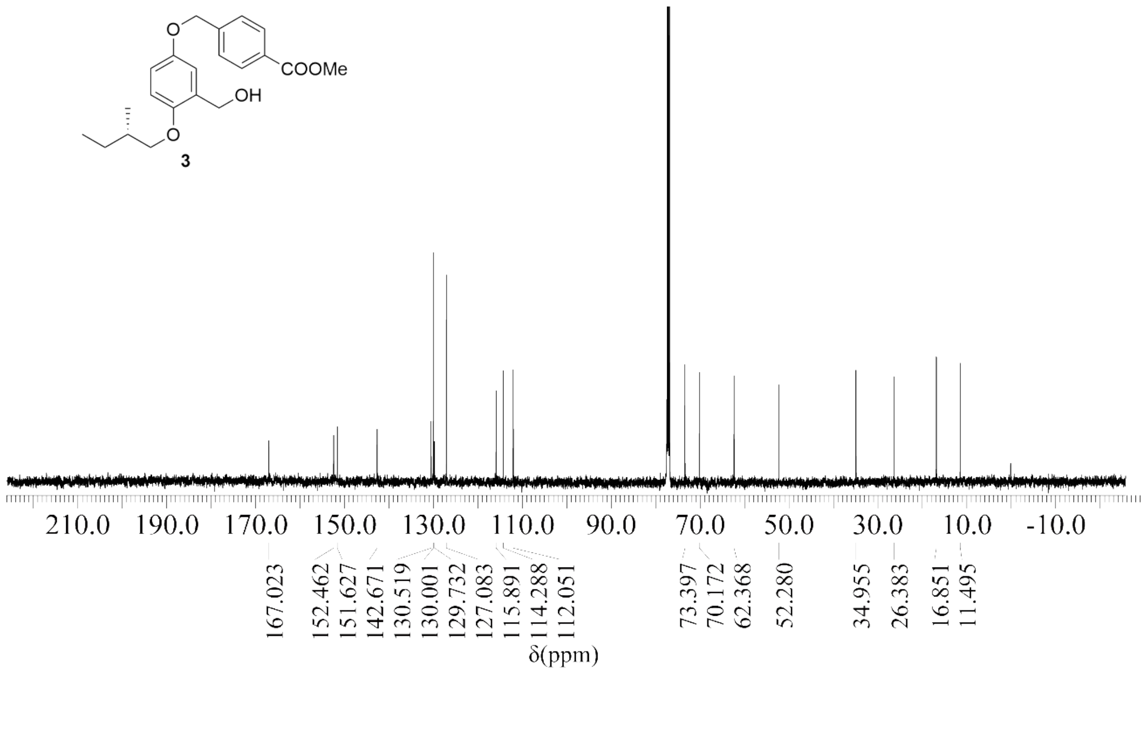
COOMe
OH
3
210.0 190.0 170.0 150.0 130.0 110.0 90.0 70.0 50.0 30.0 10.0 -10.0
167.023
152.462
151.627
142.671
130.519
130.001
129.732
127.083
115.891
114.288
112.051
73.397
70.172
62.368
52.280
34.955
26.383
16.851
11.495
δ(ppm)

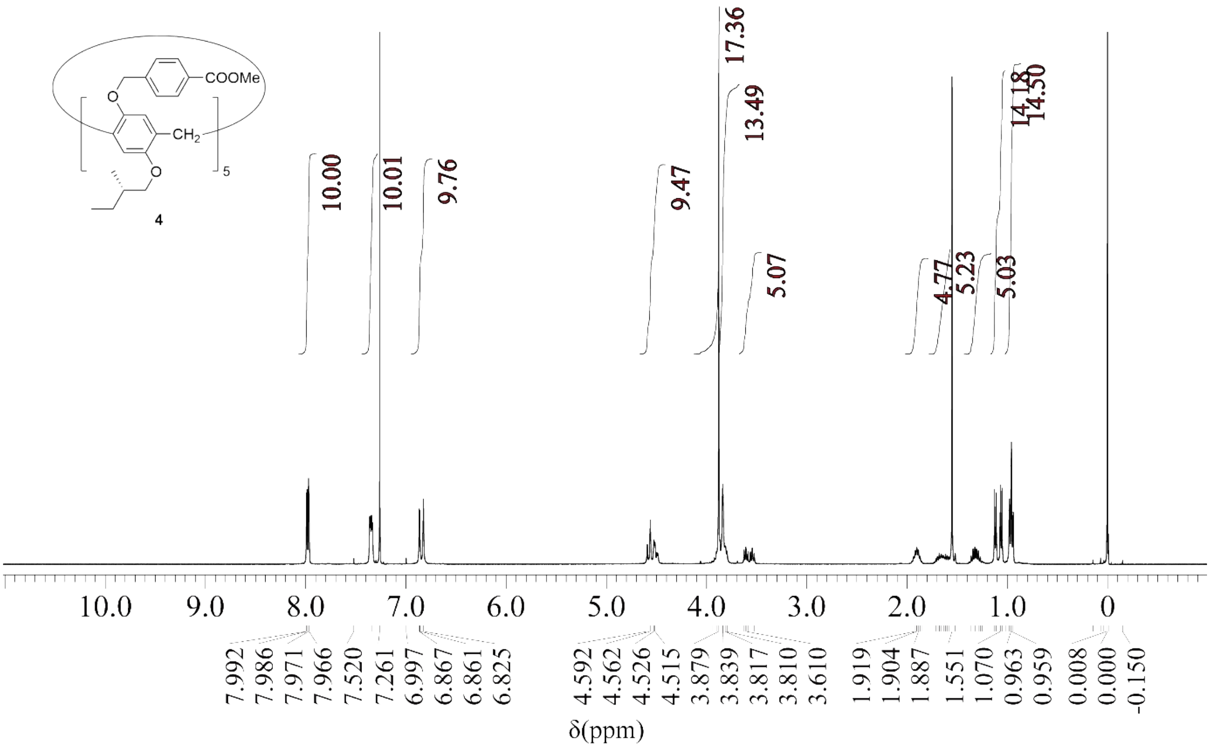
COOMe
CH2
5
4
10.00
10.01
9.76
9.47
17.36
13.49
5.07
4.77
5.23
5.03
14.18
14.50
10.0 9.0 8.0 7.0 6.0 5.0 4.0 3.0 2.0 1.0 0
7.992 7.986 7.971 7.966 7.520 7.261 6.997 6.867 6.861 6.825
4.592 4.562 4.526 4.515 3.879 3.839 3.817 3.810 3.610
1.919 1.904 1.887 1.551 1.070 0.963 0.959 0.008 0.000 -0.150
δ(ppm)

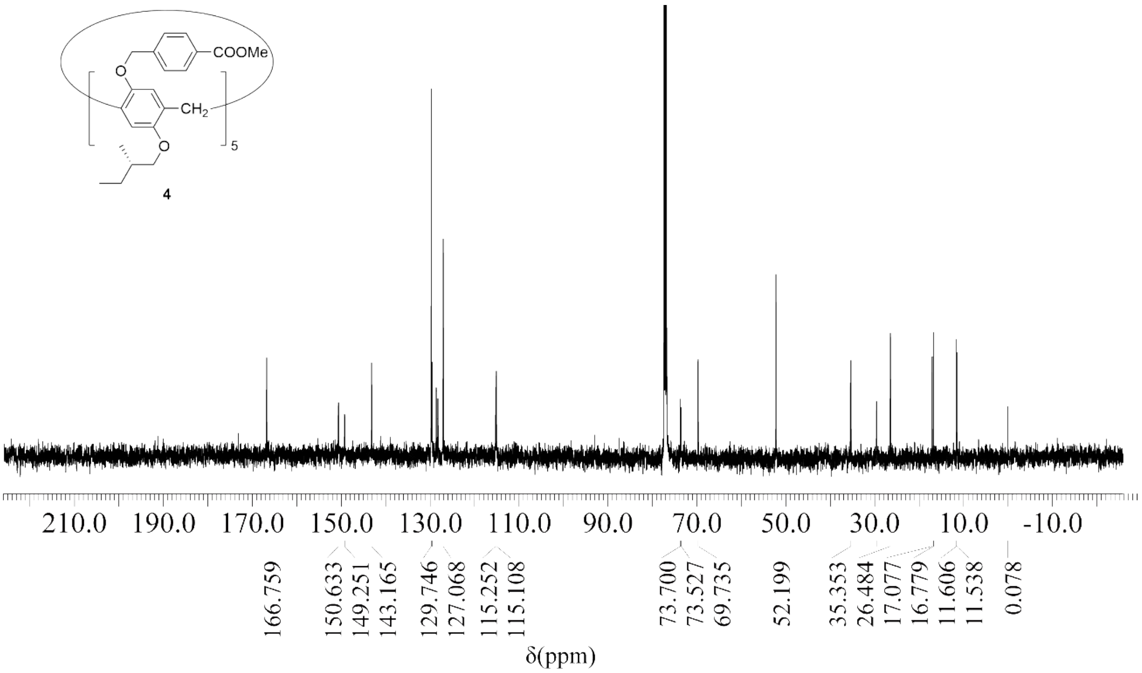
COOMe
CH2
5
4
210.0 190.0 170.0 150.0 130.0 110.0 90.0 70.0 50.0 30.0 10.0 -10.0
166.759
150.633 149.251 143.165
129.746 127.068
115.252 115.108
73.700 73.527 69.735
52.199
35.353 26.484 17.077 16.779 11.606 11.538
0.078
δ(ppm)

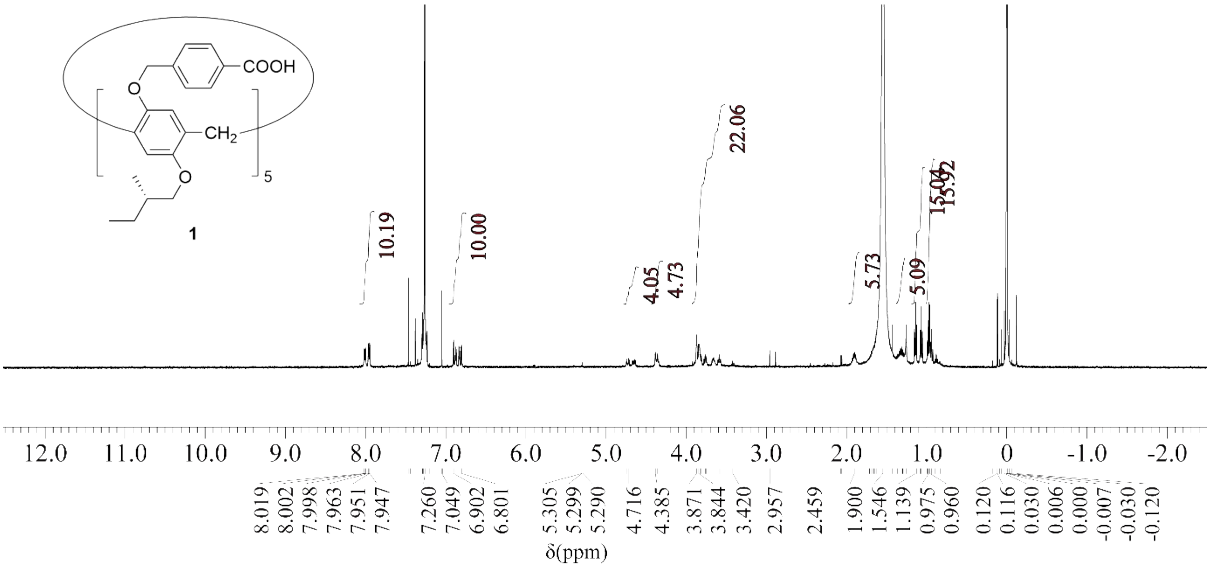

COOH
O
CH2
O
5
1
10.19
10.00
4.05
4.73
22.06
5.73
5.09
15.04
15.92
12.0 11.0 10.0 9.0 8.0 7.0 6.0 5.0 4.0 3.0 2.0 1.0 0 -1.0 -2.0
8.019 8.002 7.998 7.963 7.951 7.947 7.260 7.049 6.902 6.801 5.305 5.299 5.290 4.716 4.385 3.871 3.844 3.420 2.957 2.459 1.900 1.546 1.139 0.975 0.960 0.120 0.116 0.030 0.006 0.000 -0.007 -0.030 -0.120
δ(ppm)

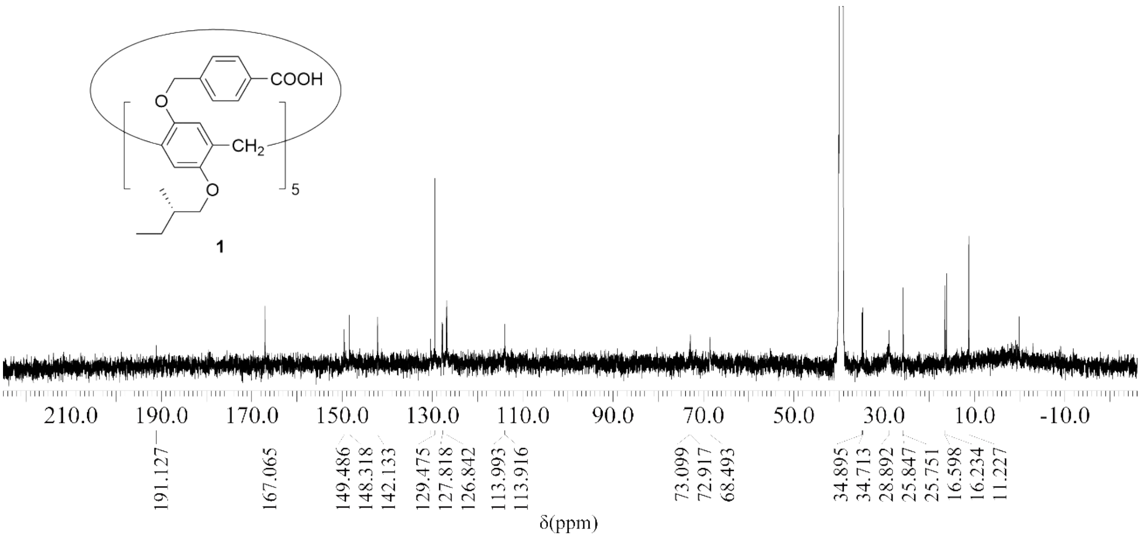

COOH
O
CH2
O
5
1
210.0 190.0 170.0 150.0 130.0 110.0 90.0 70.0 50.0 30.0 10.0 -10.0
191.127 167.065 149.486 148.318 142.133 129.475 127.818 126.842 113.993 113.916 73.099 72.917 68.493 34.895 34.713 28.892 25.847 25.751 16.598 16.234 11.227
δ(ppm)

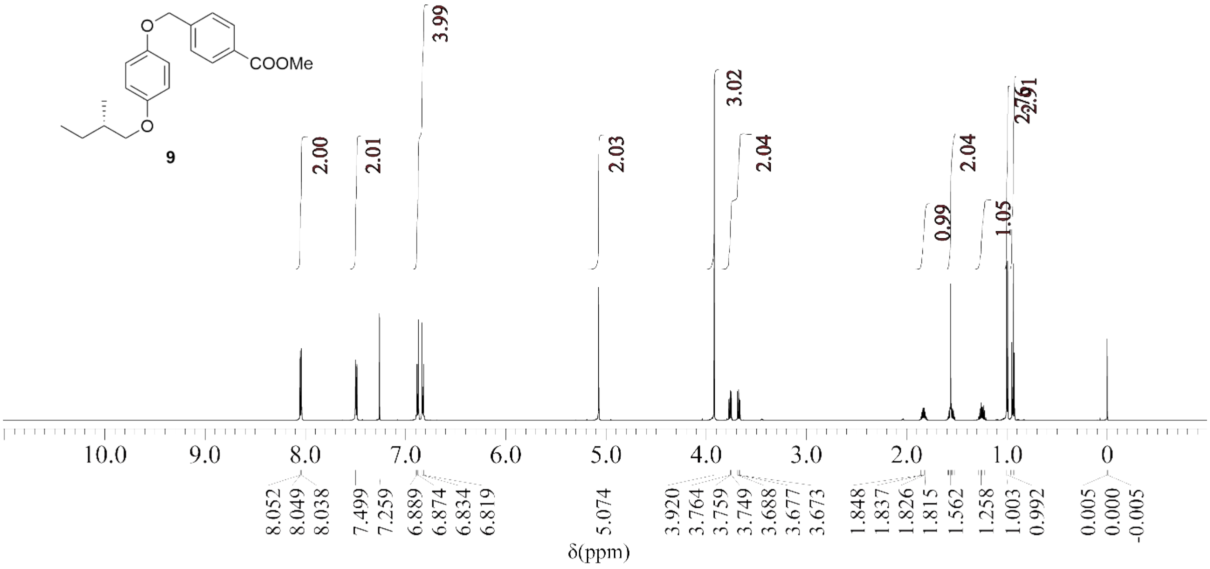
COOMe
9
2.00
2.01
3.99
2.03
3.02
2.04
0.99
2.04
1.05
2.76
2.91
10.0 9.0 8.0 7.0 6.0 5.0 4.0 3.0 2.0 1.0 0
8.052 8.049 8.038 7.499 7.259 6.889 6.874 6.834 6.819 5.074 3.920 3.764 3.759 3.749 3.688 3.677 3.673 1.848 1.837 1.826 1.815 1.562 1.258 1.003 0.992 0.005 0.000 -0.005
δ(ppm)

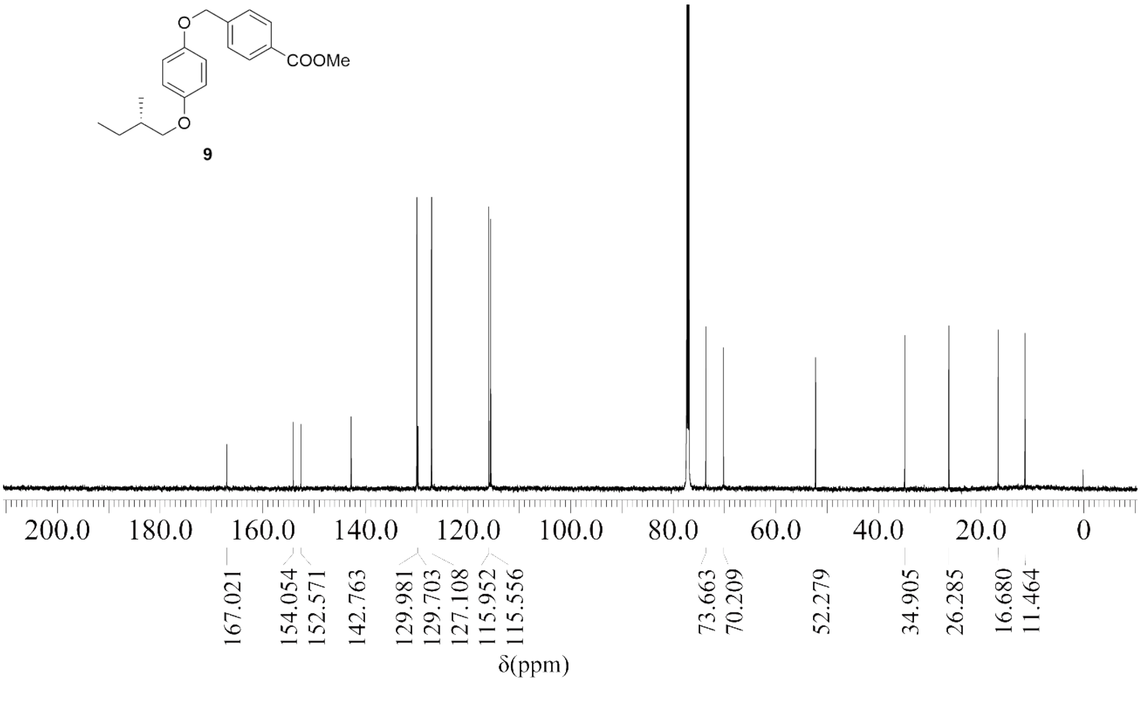
COOMe
9
200.0 180.0 160.0 140.0 120.0 100.0 80.0 60.0 40.0 20.0 0
167.021 154.054 152.571 142.763 129.981 129.703 127.108 115.952 115.556 73.663 70.209 52.279 34.905 26.285 16.680 11.464
δ(ppm)

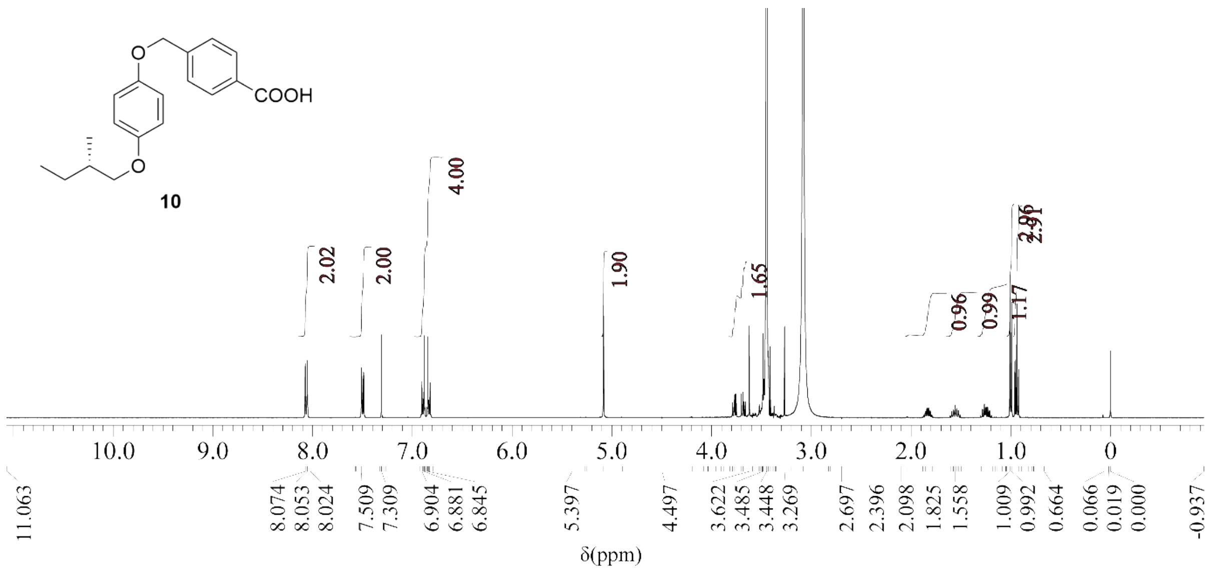
COOH
10
2.02
2.00
4.00
1.90
1.65
0.96
0.99
1.17
2.91
10.0
9.0
8.0
7.0
6.0
5.0
4.0
3.0
2.0
1.0
0
11.063
8.074
8.053
8.024
7.509
7.309
6.904
6.881
6.845
5.397
4.497
3.622
3.485
3.448
3.269
2.697
2.396
2.098
1.825
1.558
1.009
0.992
0.664
0.066
0.019
0.000
-0.937
δ(ppm)

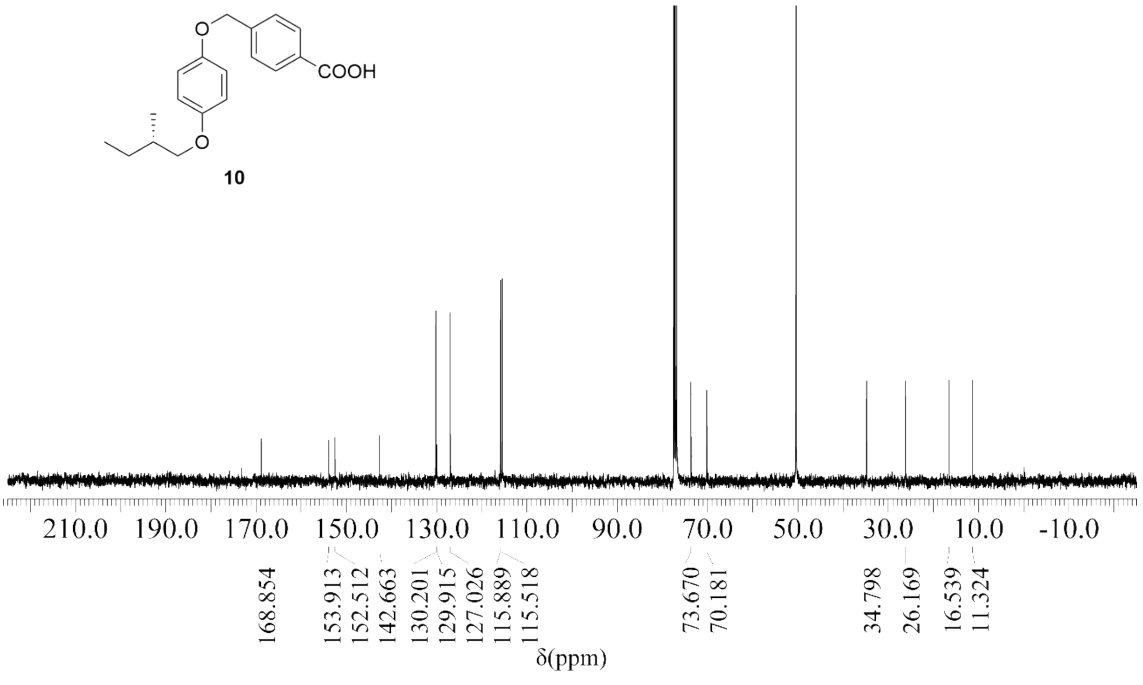
COOH
10
210.0
190.0
170.0
150.0
130.0
110.0
90.0
70.0
50.0
30.0
10.0
-10.0
168.854
153.913
152.512
142.663
130.201
129.915
127.026
115.889
115.518
73.670
70.181
34.798
26.169
16.539
11.324
δ(ppm)
